# Genome-wide identification of BAM genes in grapevine (*Vitis vinifera* L.) and ectopic expression of *VvBAM1* modulating soluble sugar levels to improve low-temperature tolerance in tomato

**DOI:** 10.1186/s12870-021-02916-8

**Published:** 2021-03-26

**Authors:** Guoping Liang, Honghong He, Guojie Nai, Lidan Feng, Yanmei Li, Qi Zhou, Zonghuan Ma, Yuan Yue, Baihong Chen, Juan Mao

**Affiliations:** 1 College of Horticulture, Gansu Agricultural University, Lanzhou, 730070 People’s Republic of China; 2 College of Food Science and Engineering, Gansu Agricultural University, Lanzhou, 730070 People’s Republic of China

**Keywords:** Grapevine, VvBAM, Low temperature, Transcriptome, qRT-PCR, Tomato

## Abstract

**Background:**

Low temperature (LT) is one of the main limiting factors that affect growth and development in grape. Increasing soluble sugar and scavenging reactive oxygen species (ROS) play critical roles in grapevine resistance to cold stress. However, the mechanism of β-amylase (BAM) involved in the regulation of sugar levels and antioxidant enzyme activities in response to cold stress is unclear.

**Results:**

In this study, six BAM genes were identified and clustered into four groups. Multiple sequence alignment and gene structure analysis showed that VvBAM6 lacked the Glu380 residue and contained only an exon. The transcript abundance of *VvBAM1* and *VvBAM3* significantly increased as temperature decreased. After LT stress, *VvBAM1* was highly expressed in the leaves, petioles, stems, and roots of overexpressing tomato lines. The total amylase and BAM activities increased by 6.5- and 6.01-fold in transgenic plants compared with those in wild-type tomato plants (WT) subjected to LT, respectively. The glucose and sucrose contents in transgenic plants were significantly higher than those in WT plants, whereas the starch contents in the former decreased by 1.5-fold compared with those in the latter under LT stress. The analysis of transcriptome sequencing data revealed that 541 genes were upregulated, and 663 genes were downregulated in transgenic plants. One sugar transporter protein gene (*SlSTP10*), two peroxidase (POD)-related genes (*SlPER7* and *SlPER5*), and one catalase (CAT)-related gene (*SlCAT1*) were upregulated by 8.6-, 3.6-, 3.0-, and 2.3-fold in transgenic plants after LT stress, respectively.

**Conclusions:**

Our results suggest that *VvBAM*1 overexpression promotes ROS scavenging and improves cold tolerance ability by modulating starch hydrolysis to affect soluble sugar levels in tomato plants.

**Supplementary Information:**

The online version contains supplementary material available at 10.1186/s12870-021-02916-8.

## Background

Plants as sessile organisms are continuously exposed to various environmental stresses [1]. Abiotic stress as universal factors can trigger many physiological, biochemical, and molecular responses that lead to various cellular processes in plants [2, 3]. Cold stress includes chilling (< 10 °C) and freezing (< 0 °C) temperatures, which are among the most crucial environmental factors that limit the geographical distribution and growth of plants [4]. Low temperature (LT) can decrease the biosynthetic activity and membrane fluidity, inhibit the normal functions of physiological and biochemical processes, causes metabolic dysfunction, and cause permanent plant damage or death in some instances [5, 6]. Therefore, improving cold hardiness in plants is an effective way to reduce the adverse effects of cold stress, increase the ability of overwintering, and cope with extreme weather events [7].

Reactive oxygen species (ROS) cause oxidative damage to cell membranes, proteins, and nucleic acids after plants are subjected to adverse environmental stresses, such as LT [8]. Under environmental stress, ROS homeostasis is broken and causes plant damage. However, plants have evolved two ways to cope with excess ROS: enzymatic and non-enzymatic antioxidant systems [9]. The enzymatic reactive system catalyzes the dismutation of O_2_^−^ into H_2_O_2_, and catalase reduces H_2_O_2_ further into H_2_O and O_2_. The other non-enzymatic system scavenges ROS via strong reducing agents, mainly including ascorbic acid (AsA) and glutathione (GSH) [10, 11]. AsA is the most abundant antioxidant substance to be increased under biotic and abiotic stress in plants. GSH also plays an important role in the antioxidant process of plants [12], and its concentration has been shown to be elevated under biotic and abiotic stresses in plants [13, 14].

Plants achieve tolerance to an adverse environment via energy metabolism and transformation. Starch as the main energy storage substance widely exists in most plants to regulate their growth and development and increase stress tolerance [15]. Beta amylase (BAM) is a typical exohydrolase, and its primary function is to degrade starch in plants [16, 17]. BAM belongs to a member of glucosyl hydrolase 14 families (GH-14) [18]. Three highly conserved sequence regions are found in all known BAM proteins. BAM has a typical glycoside hydrolase domain (PF01373) at the N terminus and contains aspartate, which is involved in the catalytic mechanism [19]. The second domain is situated at a more central location, centered around a glutamate, which is also involved in the catalytic mechanism [15]. In addition, the crystal structure of the β-amylase–maltose complex shows that the active site residues Glu186 and Glu380 are strictly conserved among BAM proteins [20]. Experimental evidence favors β-amylolysis as the main source of maltose during starch breakdown, producing maltose [17]. The deficiency of BAM may reduce the ability to degrade starch in plants, especially in the dark, indicating that starch hydrolysis plays an important role in the adaptation of plants to unfavorable environments [21]. Starch degradation is dependent on BAM activity in *A. thaliana* and other organisms [22]. Kaphlan et al. [23] reported that BAM1 accounts for more than 90% of the total BAM activity in the mesophyll cells of *A. thaliana*. Li et al. [24] demonstrated that *AtBMY7* (At3g23920) is induced by high temperature to encode a protein with a putative chloroplast transit peptide in *A. thaliana*. This protein can respond to heat shock. Thalmann et al. [25] found that starch is rapidly mobilized by the synergistic action of *AtBAM1* and *AtAMY3* to promote stomatal opening. Universally, AtBAM1 catalyzes starch degradation in the dark. However, AtBAM1 can also catalyze starch degradation during the day when plants are under stress [25].

Gene expression and protein activity are induced by light [26], LT [27], drought and salt [28, 29], and osmotic stress [30]. BAM transcript expression and activity are induced during temperature stress, and an increase in maltose content is associated with BAM transcript and activity [31, 32]. Monroe et al. [22] reported that BAM3 contributes to leaf starch degradation in mesophyll cells at night and under cold stress. PtrBAM1 (α- and β-amylase-coding gene of *Poncirus trifoliata*) overexpression increases the BAM activity in tobacco leaves and promotes the accumulation of maltose and soluble sugar under cold stress [33]. Zhang et al. [34] found that LT can induce high transcription of StBAM7 and StBAM9 in potato tubers but not in other tissues. Prasch et al. [29] demonstrated that AtBAM1 regulates starch degradation in guard cells and affects the stomatal opening of *A. thaliana* subjected to drought stress; however, the impaired starch breakdown of *bam1* mutant plants is accompanied with reduced stomatal opening. Moreover, *bam1* mutants are impaired in proline accumulation and suffer from stronger lipid peroxidation than WT plants [30].

Grapevine (*Vitis vinifera* L.) is a widely cultivated fruit crop with a high economic value. However, its biological characteristic of LT sensitivity limits cultivation and development. *Vitis amurensis* is an extremely cold-resistant grapevine species that can withstand LT down to − 40 °C [35]. Therefore, it is used as a source to isolate target genes to be used to introduce cold tolerance in various crop plants. Tomatoes originate in tropical and subtropical areas. They are sensitive to LT, so they serve as good model plants for studying functional genes related to LT. In this work, a LT-responsive gene (*VvBAM1*) was cloned and overexpressed in tomato plants. *VvBAM1* overexpression in tomato afforded apparent tolerance against cold stress.

## Results

### Evolutionary analysis of BAM proteins from different plant species

**Fig. 1:**
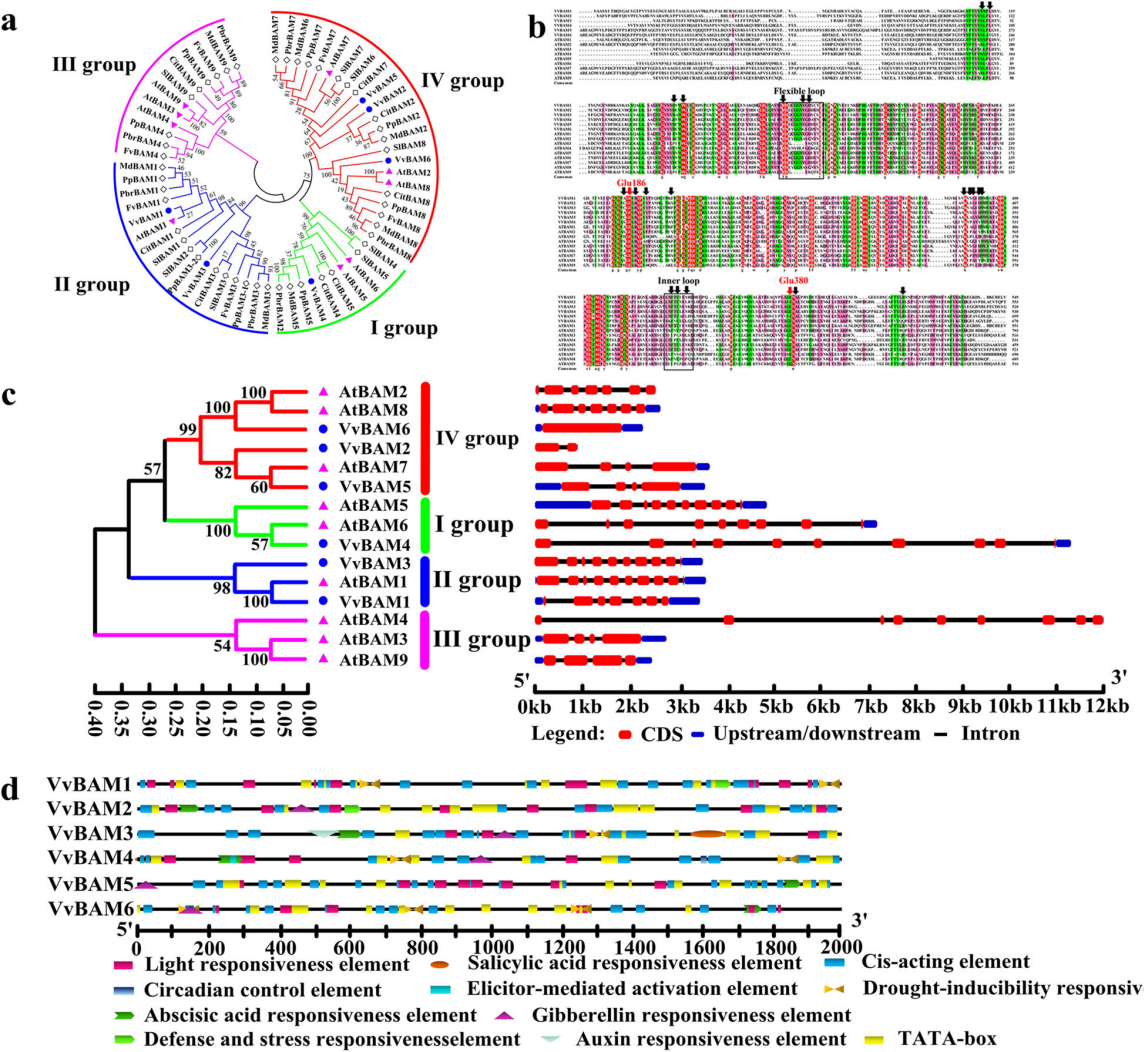
Bioinformatics analyze VvBAM genes family. **a** Phylogenetic evolution of VvBAM genes family. Red for IV group, green for I group, blue for II group, and pink for III group. Pink triangles for grape BAM, blue cycles for Arabidopsis BAM. These colors and symbols also apply to the (**c**). **b** Multiple sequence alignment of VvBAM and AtBAM amino acid, black arrowheads indicate substrate-binding residues, and red arrowheads represent the two catalytic residues (Glu186 and Glu380). **c** Gene structure analysis of *VvBAM* and *AtBAM*. **d** *Cis*-elements analysis of VvBAM gene family

Six BAM genes (*VvBAM1*–*6*) were retrieved from the Grape Gene Database (Table 1). A phylogenetic tree was constructed to analyze the evolutionary relationship of 62 BAM amino acid sequences from grape, apple, citrus, pear, peach, strawberry, and tomato (Fig. 1a, Supplementary Table S2). These genes were divided into groups I, II, III, and IV via reference to the evolution of BAM genes in *A. thaliana*. *VvBAM4* was in group I, *VvBAM1* and *VvBAM3* were clustered into group II, *VvBAM2*, *VvBAM5*, and *VvBAM6* were clustered into group IV, and no grapevine BAM gene member was clustered into group III. The 15 motif modules were used to map the BAM gene families of the eight species. Six gene members of grapevine BAM had 15 motif modules (Supplementary Fig. S1). The BAM gene family was distributed on four chromosomes (Table 1). *VvBAM1* and *VvBAM4* were located on the 5th and 12th chromosomes, respectively; *VvBAM2* and *VvBAM5* were found on the 15th chromosome; *VvBAM3* and *VvBAM6* were detected on the 2nd chromosome. The number of amino acids ranged from 543 (VvBAM3) to 699 (VvBAM5). The molecular weights of these proteins were between 6.05 (VvBAM3) and 7.88 (VvBAM5) kD. The predicted isoelectric point (pI) was 5.25–5.86 (Table 1). The conserved protein sequences of the glycoside hydrolase domains of grapevine were aligned with those of the *A. thaliana* enzyme AtBAM (Fig. 1b). The multisequence alignment of grapevine and *A. thaliana* BAM amino acids revealed that Glu186 was highly conserved in both species, whereas Glu380 was deleted in the VvBAM6 sequence. The gene structure showed that *VvBAM3* and *VvBAM*4 contained 10 exons and 9 introns, respectively (Fig. 1c). *VvBAM2*, *VvBAM3*, and *VvBAM4* had no 5′ untranslated region (UTR), but *VvBAM1*, *VvBAM5*, and *VvBAM6* had 5′ and 3′ UTRs. The gene sequence of *AtBAM4* was the longest, followed by that of *VvBAM4*. The gene sequence of *VvBAM2* was the shortest. The promoter sequences of these genes were composed of unequal amounts of light-, defense-, and hormone-responsive elements, such as those for gibberellin, abscisic acid, salicylic acid, and auxin (Fig. 1d).

**Table 1:** The physical and chemical properties of VvBAMs proteins

Gene name	Gene accession NO.	Chromosome location	Full length (bp)	Amino acid residue	*pI*	Molecular weight (kD)	Arabidopsis homologous gene	
							Gene accession NO.	Gene name
*VvBAM1*	GSVIVT01001863001	Chr.05: 162431..173208	10,778	573	5.86	6.31	AT3G23920	*AtBAM1*
*VvBAM2*	GSVIVT01026920001	Chr.15: 19379225..19386528	7304	554	5.66	6.26	AT4G00490	*AtBAM2*
*VvBAM3*	GSVIVT01013272001	Chr.02: 5804013..5807077	3065	543	8.71	6.05	AT4G15090	*AtBAM3*
*VvBAM4*	GSVIVT01030642001	Chr.12: 7374750..7377610	2861	596	5.25	6.63	AT2G32290	*AtBAM6*
*VvBAM5*	GSVIVT01026922001	Chr.15: 19389359..19399834	10,476	699	5.58	7.88	AT2G45880	*AtBAM7*
*VvBAM6*	GSVIVT01036911001	Chr.02: 17760098..17774855	14,758	670	5.59	7.53	AT5G45300	*AtBAM8*

### Expression profiles of *VvBAM* genes under different growth periods in the phloem of grapevine

**Fig. 2:**
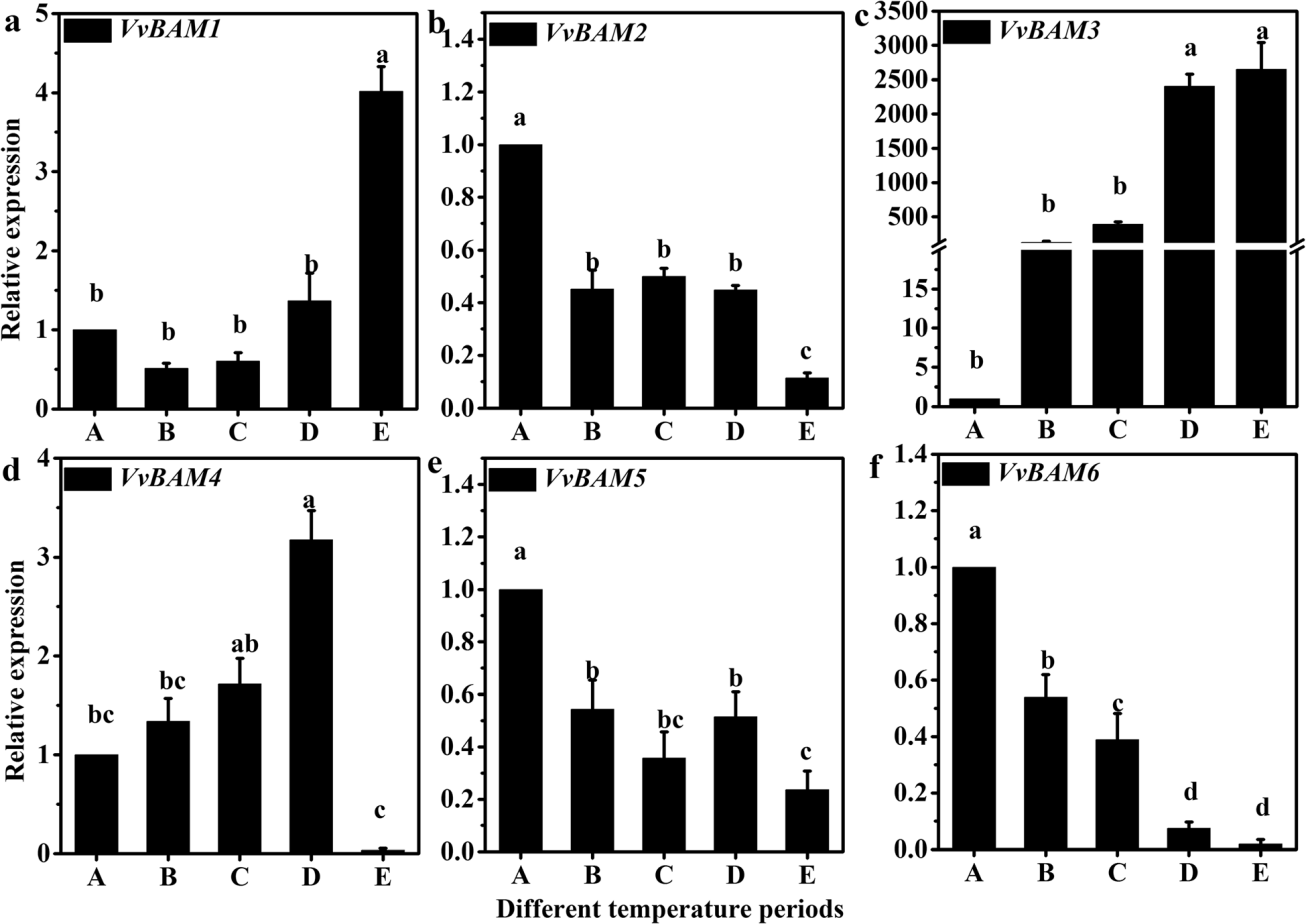
Expression analysis of the *VvBAM1* – *VvBAM6* gene family in grape phloem subjected to different LT stress

The results of quantitative real-time polymerase chain reaction (qRT-PCR) showed that the six *VvBAM* genes had different expression patterns during the LT dormancy. The relative expression of *VvBAM1* increased as temperature decreased, especially in the E period (Fig. 2a). The relative expression of *VvBAM3* also increased remarkably as temperature decreased from A to E period (Fig. 2c). However, *VvBAM2*, *VvBAM5*, and *VvBAM6* were substantially downregulated as temperature decreased (Fig. 2b, e, and f). Only the expression of *VvBAM4* increased from A to D period but decreased considerably in the E period (Fig. 2d). These results could provide a solid foundation for identifying the function of VvBAM*.*

### Differential expression profiles of *VvBAM1* in diverse tissues of transgenic tomato plants under LT

**Fig. 3:**
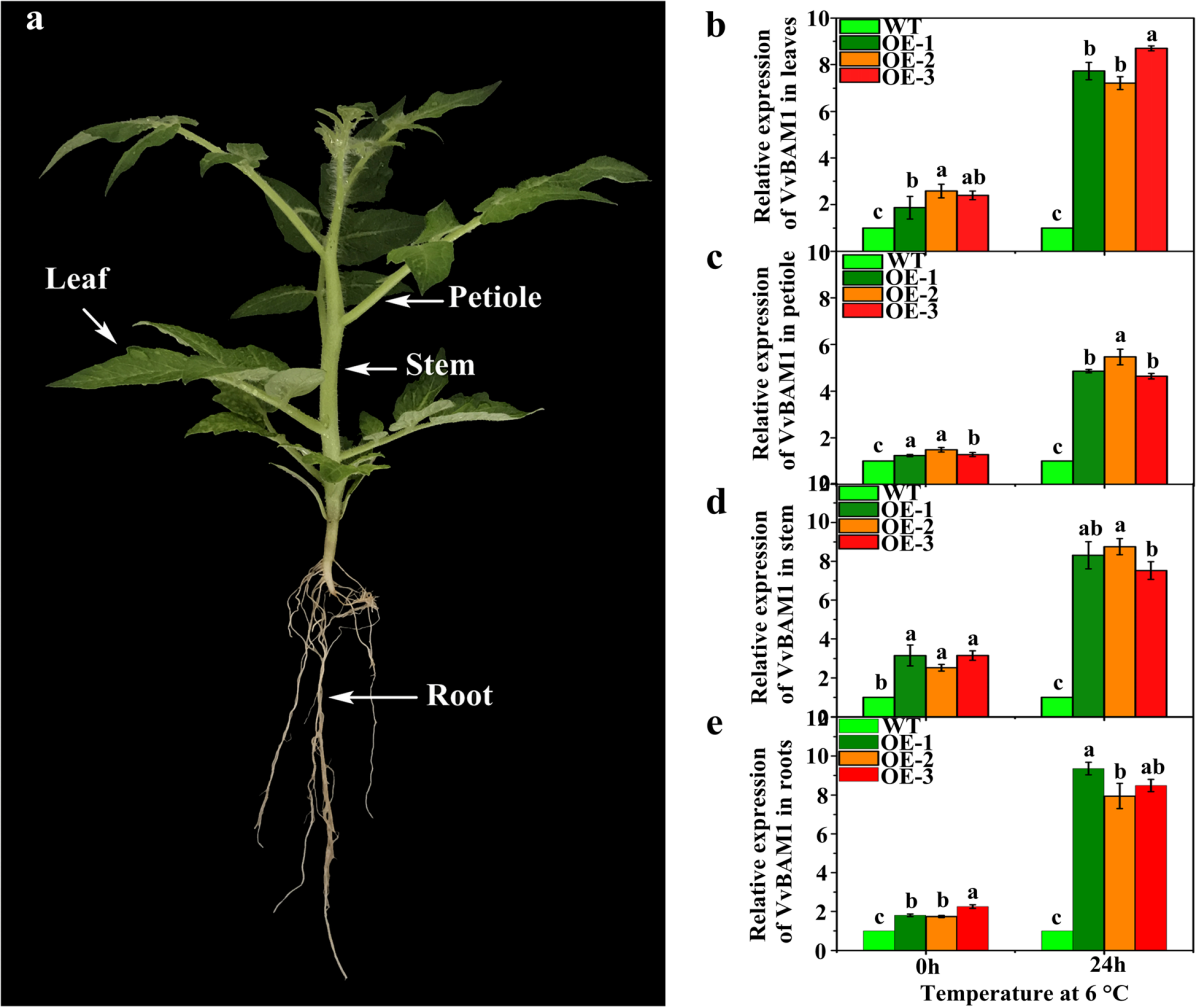
Tissue specific expression of *VvBAM1* in WT and transgenic plants were subjected LT stress. **a** Sample position for tomato tissue-specific detection. **b** – **e** Relative expression analysis of *VvBAM1* in leaves, petioles, stems, and roots

The relative expression level of *VvBAM1* significantly differed in WT and transgenic plants in different tissue organs. Under the no-stress condition, the expression of *VvBAM1* in the transgenic plant leaves was significantly higher than that in the WT plant leaves (Fig. 3). After LT for 24 h at 6 °C, the relative expression levels of *VvBAM1* were significantly upregulated by 7.7-, 7.2-, and 8.7-fold and remarkably higher in the transgenic plant leaves than in the WT plant leaves at 0 h (Fig. 3b). The expression level of *VvBAM1* was significantly higher in the transgenic plant petioles than in the WT plant petioles under no-stress and stress conditions (Fig. 3c). The relative expression of *VvBAM1* in transgenic plant stems was substantially higher than that in WT plant stems under no stress and after LT stress (Fig. 3d). The expression trend of *VvBAM1* in the roots was consistent with that in the leaves, petioles, and stems (Fig. 3e).

### Phenotypic and staining analysis of transgenic tomato plants under LT condition

**Fig. 4:**
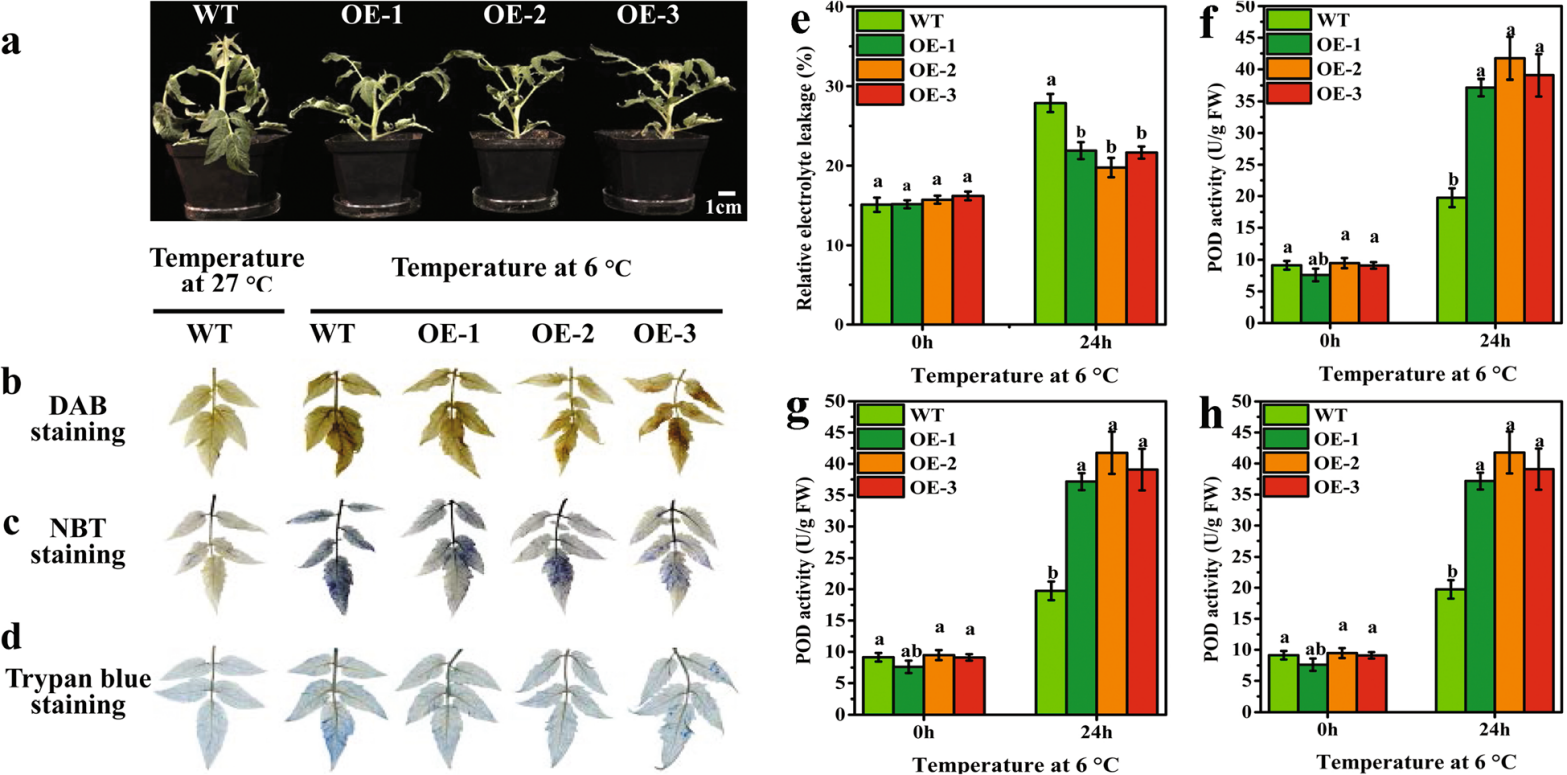
The analysis of reactive oxygen content and antioxidant enzyme activity in WT and transgenic plants under LT stress at 6 °C for 24 h. **a** Phenotype changes under the LT treatment of 6 °C for 24 h. **b** – **d** Detection of H_2_O_2_, O_2_^−^ and injured cells by DAB, NBT, and trypan blue staining in WT and transgenic plants. **e** – **h** Analysis of relative electrolyte leakage, SOD, POD, and CAT activity in leaves

The height and root length of the WT plants were remarkably higher than those of the transgenic plants (Supplementary Figs. S3a, b, and S4). The WT and transgenic plants were subjected to gradient temperature adaptation and stress at 6 °C for 72 h, and the relative electrolyte leakage and *VvBAM1* expression levels were selected to determine the parameters for 24 h of stress (Supplemental Figs. S5, S6a, and c). The results showed that the degree of the wilting of the leaves of the transgenic plants was less than that of the WT plants after LT stress for 24 h (Fig. 4a). DAB, NBT, and trypan blue staining was performed to detect H_2_O_2_ accumulation, O_2_^−^ accumulation, and cell damage levels in the leaves, respectively. The H_2_O_2_ accumulation was not obvious at 27 °C in the WT tomato leaves (Fig. 4b). After LT stress at 6 °C for 24 h, the leaves of three transgenic plants turned brown, but the color was slightly lighter than the WT leaves. The O_2_^−^ accumulation showed that the stained area of the leaves of WT plants after LT stress was obvious, but the stained area of the transgenic plant leaves after LT stress was not evident (Fig. 4c). Trypan blue staining revealed that the stained area of the transgenic tomato plants was lighter than that of the WT plants under LT stress, whereas the staining area of the WT plants under normal growth conditions was lighter than that under LT stress (Fig. 4d). These results indicated that transgenic tomato plant leaves had low ROS contents.

### Effects of ectopic *VvBAM1* expression on antioxidant enzyme activities in tomato plants under LT stress

The leaf staining of WT and transgenic tomato plants showed that the accumulation of H_2_O_2_ (Fig. 4b) and O_2_^−^ (Fig. 4c) differed after LT stress. Therefore, the relative electrolyte leakage, POD, SOD, and CAT in the leaves of WT and transgenic plants were measured to evaluate antioxidant enzyme activities. The relative electrolyte leakage indicated that the WT and transgenic plants following LT stress were considerably higher than those under normal growth conditions; however, the transgenic plants were remarkably lower than that of WT (Fig. 4e). Generally, ROS accumulation when the plants are subjected to environmental stress, however, the antioxidant systems of plant help to counter up oxidative stress generated ROS [36]. Hence, the activities of POD, SOD, and CAT were measured. The results showed that the POD activity between WT and transgenic plants under normal growth conditions was not significantly different; however, it significantly increased by 1.88-, 2.11-, and 1.98-fold in transgenic plants after 24 h of LT stress at 6 °C (Fig. 4f). The SOD activity was not significantly different between WT and transgenic plants under normal growth conditions, but its activity remarkably increased after 24 h of LT stress. Its activity in the transgenic plants was significantly higher than that in the WT plants (Fig. 4g). The trend of the CAT activity was consistent with that of the POD and SOD activities after LT stress (Fig. 4h). The CAT activity significantly increased by about 1.16-fold in the transgenic plants after LT stress compared with that in the WT plants. These data demonstrated that *VvBAM1* overexpression could significantly promote O_2_^−^ and H_2_O_2_ scavenging via antioxidant enzymes.

### Difference in amylase activities and soluble sugar contents in transgenic tomato plants under LT stress

**Fig. 5:**
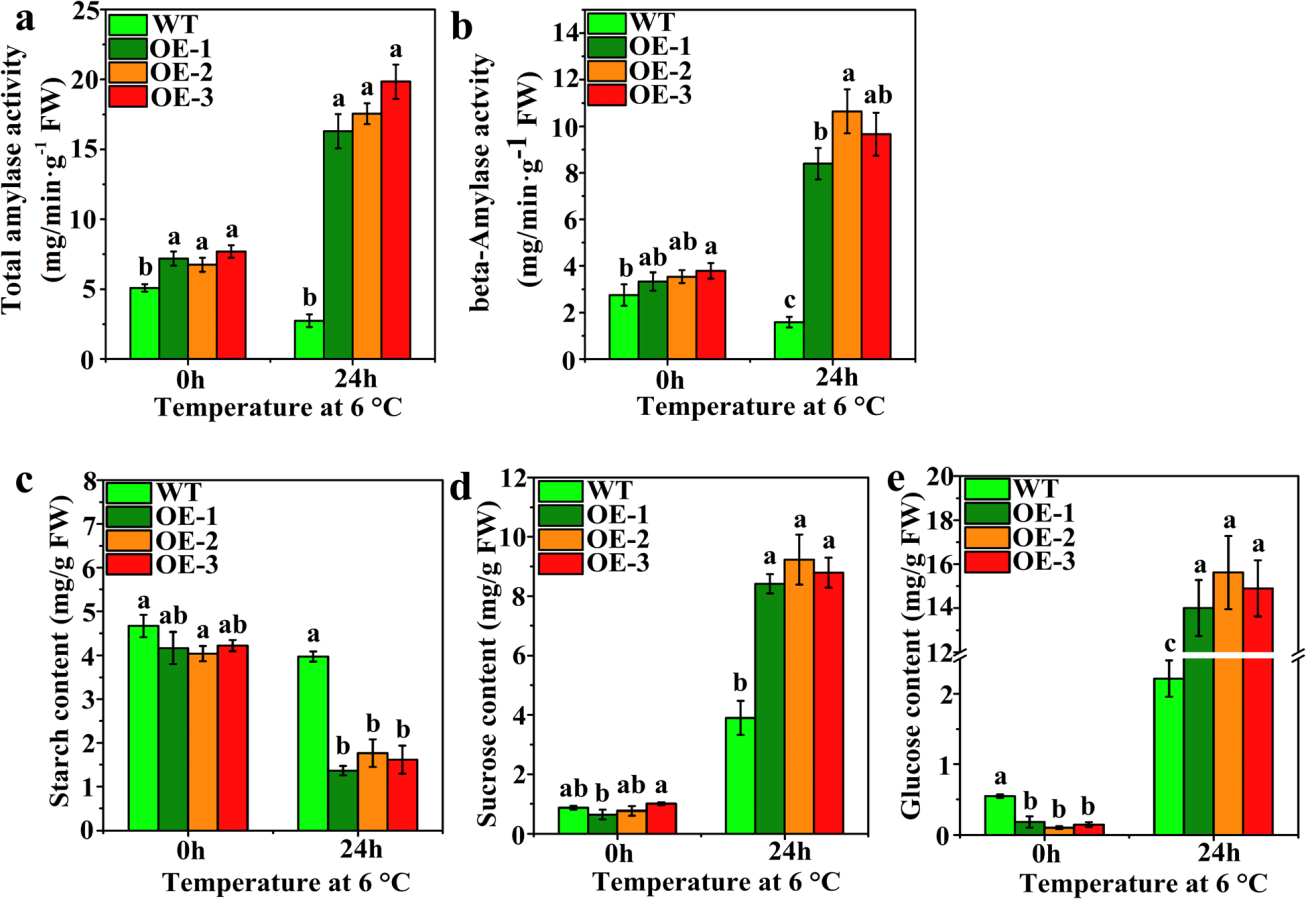
Changes of amylase activity and soluble sugar content in WT and transgenic plants. **a**, **b** The changes of total amylase and BAM activity. **c** – **e** The changes of starch, sucrose, and glucose levels

**Fig. 6:**
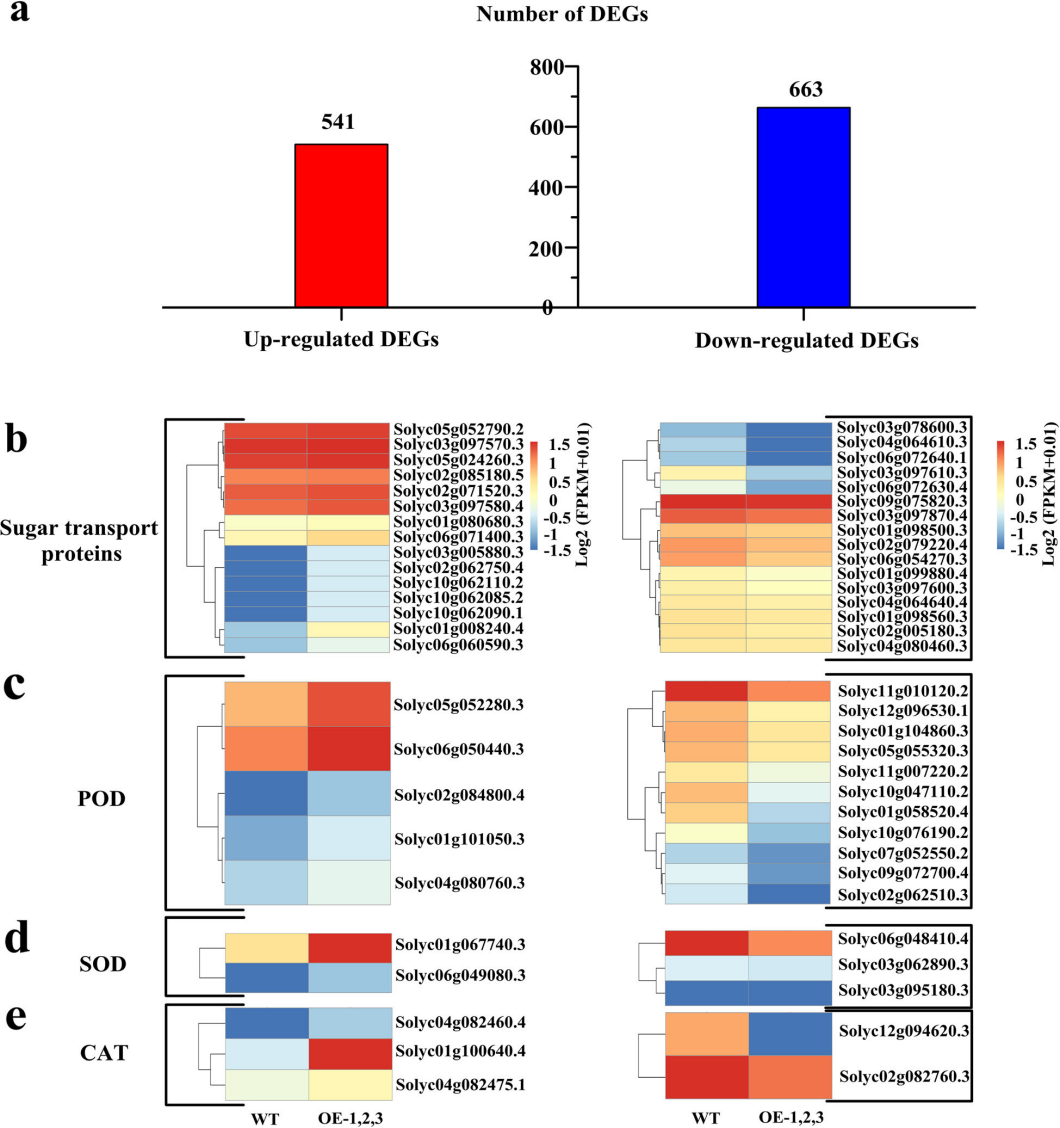
Transcriptome sequencing analysis of DEGs. **a** DEGs of up- and down-regulation in leaves of WT and transgenic plants following LT stress for 24 h. **b** Heatmap analysis of DEGs were related to sugar transport protein, POD, SOD and CAT in response to LT stress

The activities of total amylase and β-amylase were determined in WT and transgenic plants under normal and LT stress conditions (Fig. 5a and b). The total amylase activity in transgenic plants was remarkably higher than that in WT plants under normal conditions and following LT stress (Fig. 5a). In addition, the total amylase activity of the WT plants was inhibited after LT stress, but the total amylase activity of the transgenic plants was significantly elevated (Fig. 5a). Under normal conditions, the β-amylase activity of the transgenic plants was significantly higher than that of the WT plants (Fig. 5b). After 24 h of LT stress, the β-amylase activities of the WT plants were reduced, but those of the transgenic plants were significantly increased. The starch, sucrose, and glucose contents were assayed in WT and transgenic plants under normal and stress conditions (Figs. 5c – 6e). The starch content had no significant difference in the WT and transgenic plants (OE-1 and OE-3) under normal conditions (Fig. 5c). After 24 h of LT stress, the starch contents of the WT and transgenic plants decreased, but the starch contents of the transgenic plants decreased more significantly than those of the WT plants. The sucrose content substantially increased after LT stress (Fig. 5d). Its content was not remarkably different between the WT and transgenic plants under normal conditions, but a considerable difference was observed between the WT plants and the three transgenic plants after the LT stress. The sucrose content of the transgenic plants was higher than that of the WT plants. The glucose content substantially increased after LT stress (Fig. 5e). The glucose content significantly differed between the WT and transgenic plants under normal growth conditions. After 24 h of LT stress, the glucose content of the transgenic plants was considerably higher than that of the WT plants, i.e., increased by 6.7-fold. These results indicated that the trend of changes in soluble sugar contents and amylase activities was consistent.

### RNA-sequencing profile analysis of the differential expression of sugar transporter and antioxidant enzyme genes in transgenic tomato plants

A total of 1204 DEGs (541 upregulated genes and 663 downregulated genes) were identified in the WT and transgenic plants via LT treatment (Fig. 6a). The sugar transport protein included 15 upregulated DEGs and 16 downregulated DEGs (Fig. 6b, Supplementary Table S4). Two sugar transport protein genes of Solyc06g071400.3 (*SlSWEET4*) and Solyc01g008240.4 (*SlSTP10*) were upregulated by 2.9- and 18.6-fold in the transgenic plants after LT stress, respectively. Solyc06g0726304.1 (*SlSWEET11*) and Solyc03g097610.3 (*SlSWEET10*) were downregulated by 23.5- and 34.8-fold in the transgenic plants. Furthermore, 5 upregulated DEGs and 11 downregulated DEGs in POD protein, Solyc02g084800.4 (*SlPER7*) and Solyc01g101050.3 (*SlPER5*) were upregulated by 3.6- and 3.0-fold, respectively; Solyc01g104860.3 (*SlPER4*) and Solyc11g010120.2 (*SlPER1*) were downregulated by 2.1- and 2.4-fold among these genes in the transgenic plants after LT stress, respectively (Fig. 6c). Two (Solyc01g067740.3 and Solyc06g049080.3) upregulated and three (Solyc03g062890.3, Solyc03g095180.3, Solyc06g048410.3) downregulated DEGs were found in SOD (Fig. 6d). The CAT protein included three upregulated DEGs and two downregulated DEGs, and Solyc01g100640.4 (*SlCAT1*) was upregulated by 2.3-fold (Fig. 6e). These results revealed that the related genes of sugar transport proteins, and POD, SOD, and CAT proteins were upregulated/downregulated responses to LT stress in transgenic tomato plants.

## Discussion

### Evolution of *BAM* genes in grapevine plants

BAM proteins modulate starch degradation to contribute to plant stress responses. Yue et al. [15] reported that *BAM* genes belong to multigene families. For instance, 4, 9, 13, and 9 *BAM* genes are found in rice, barley, banana, and *A. thaliana*, respectively [23, 37–39]. In this study, six genes encoding BAM (*VvBAM1*–*6*) were obtained from the grapevine genome database. These BAM proteins were classified into four families (Fig. 1a). The phylogenetic tree of *A. thaliana*, peach, tomato, apple, citrus, strawberry, and pears indicated that BAM proteins were evolutionarily conserved in higher plants. BAM belongs to the GH-14 protein with a conserved core glycoside hydrolase domain. Soybean GmBAM1 and sweet potato StBAM1 were analyzed through X-ray crystallography, and the results demonstrated that BAM has a substrate-binding pocket and active site [40, 41]. Substrate binding was accompanied with movements of an inner loop and a flexible outer loop, and catalysis was mediated by a pair of conserved glucose residues [17]. Multiple sequence alignment with nine AtBAM proteins from *A. thaliana* revealed that the VvBAM6 peptide lacked a Glu380 amino acid catalytic residue (Fig. 1b), and *VvBAM6* showed a downward regulation expression trend under different temperature periods in grapevine (Fig. 2f). This result is consistent with the previously reported observations of Kaplan et al. [21] in the expression trend of *AtBAM7* under LT stress because *VvBAM6* and *AtBAM7* were clustered into group IV in the phylogeny (Fig. 1a and c). The gene structure showed that *VvBAM2*, *VvBAM3*, and *VvBAM4* did not contain 5*′* UTRs possibly because of the incompleteness of the grapevine genome database (Fig. 1c). Interestingly, *VvBAM2*, *VvBAM5*, *VvBAM6*, and *AtBAM7* were clustered in group IV, and these genes were downregulated after LT stress (Fig. 1a and c). These genes might have a similar biochemical function in plants in response to LT stress [21]. The transcription initiation site upstream the 2 k bp sequence of all the *VvBAM* genes was analyzed to determine LT-related elements. The results showed that all of *VvBAM1*–*6* contained a light responsive element, and the promoter of *VvBAM1* had two drought responsive elements and one defense- and stress-responsive element (Fig. 1d) [34, 42]. These elements might have caused a VvBAM1 response to LT stress.

### *VvBAM1* overexpression improves the LT tolerance of tomato plants by promoting ROS scavenging

Abiotic stresses, such as drought, salinity, and LT, often produce a high amount of ROS in plant photosynthetic tissues [8]. ROS accumulation can change the mechanism of photosynthesis, resulting in photoinhibition [43]. The main downstream targets of ROS during oxidative stress are nucleic acids, proteins, and lipids [44]. Cell membranes are considered the primary target, and their stability and integrity are of primary concern for plants grown under abiotic stress conditions [45]; however, most plants in adverse environments are prone to cell membrane damage [46]. Plants have evolved various mechanisms, such as antioxidant enzymes, to scavenge ROS, and some of the reductive substances detoxify harmful ROS [47, 48]. Moreover, sugar can serve as osmotic regulatory agents to participate ROS scavenging. Shen et al. [49] proposed that the increased endogenous sugar content is not only regulated the osmotic potential in cells to improve plant cold tolerance, also which via other paths to reinforce cold tolerance, such as ROS scavenging. BAM1 protein was activated via thioredoxin-mediated reduction [50], and this mechanism is associated with the change in stromal redox potential driven by the photosynthetic electron transport chain via the electron carrier ferredoxin [17, 51]. Therefore, the BAM1 protein can contribute to scavenge excess ROS in chloroplasts. In this study, DAB and NBT stains on the transgenic plant leaves were lighter than those on the WT plant leaves under LT stress. This result showed that the H_2_O_2_ and O_2_^−^ accumulation levels of the transgenic plants were lower than that of the WT plants. This phenomenon contributed to the protection of the photosynthetic electron transport chain during cold stress [23]. Our results also showed that the ectopic VvBAM1 expression significantly increased the antioxidant enzyme activities (POD, SOD, and CAT) in transgenic plants (Fig. 4f, g, and h), whereas the ROS (H_2_O_2_ and O_2_^−^) contents and electrolyte leakage decreased under LT stress (Fig. 5b, c, and d). These results were consistent with the results of Guy and Kaplan [23]. Moreover, the related genes of the antioxidant enzymes were upregulated in the transgenic tomato plants under LT stress (Fig. 6c, d, and e). These results indicated that ROS were scavenged because of the VvBAM1-regulated expression of antioxidase-related genes in transgenic tomato plants under LT stress.

### *VvBAM1* increases LT tolerance by altering amylase activities and modulating sugar levels

Most plants encounter different biotic and abiotic overlapping or sequential stress under natural growth conditions. However, stress triggers plants to adapt to unsuitable environments and reproduce [52]. Carbohydrate metabolism plays a major role in plant metabolism, providing energy for the growth and development of plants [53]. Photosynthesis is the fundamental biological process that converts inorganic carbon into organic carbon from solar radiant energy [54]. Therefore, photosynthesis has become the main plant energy source that ensures plants can survive in the stress environment. A large fraction of the carbon fixed by photosynthesis is stored in chloroplasts with the form of starch and then degraded at night. BAMs participate in the breakdown of leaf starch [17]. Starch is hydrolyzed by BAMs to form soluble sugars, which are crucial osmoregulation substances during the life cycle of higher plants. BAM transcription and activities can be induced by LT, and this result is associated with an increase in soluble sugar contents [23]. Another aspect that can contribute to tolerance capacity under LT stress is carbon partitioning, which involves sugar metabolism and energy production [52]. Under chilling stress, the leaves of chilling-tolerant maize plants had less starch and higher sucrose-to-starch ratio than the leaves of chilling-sensitive maize plants [27]. The leaves of cold-tolerant majority plants, which contain a large amount of sucrose under chilling stress, can maintain membrane integrity [55]. Our results showed that starch the content was greatly reduced in the leaves of transgenic tomato plants under LT stress, glucose and sucrose contents increased, and the transgenic plants slightly wilted (Figs. 4a, e, 5c, d, and e). These results indicated that *VvBAM1* overexpression could enhance the cold resistance of transgenic tomato plants. Storm et al. [56] demonstrated that BAM3 enzymatic activity decreases in cold-stressed *A. thaliana* leaves, whereas the BAM1 enzymatic activity was largely unaffected. Further studies have indicated that BAM3 is being inhibited by post-translational modification because of excessive starch accumulation under cold stress. Peng et al. [33] suggested that *PtrBAM1* overexpression in tobacco increased the BAM activity and soluble sugar accumulation under cold stress. In the present work, the total amylase and β-amylase activities were remarkably higher in the *VvBAM1*-overexpressing transgenic plants than in the WT plants after LT stress (Fig. 5a and b). The tissue-specific expression showed that the expression of *VvBAM1* in the transgenic plants was markedly higher than that in the WT plants. Therefore, the starch was converted soluble sugars, which not only provided energy for tissues but also participated in osmotic regulation under LT stress (Fig. 3a) [33, 57].

**Fig. 7:**
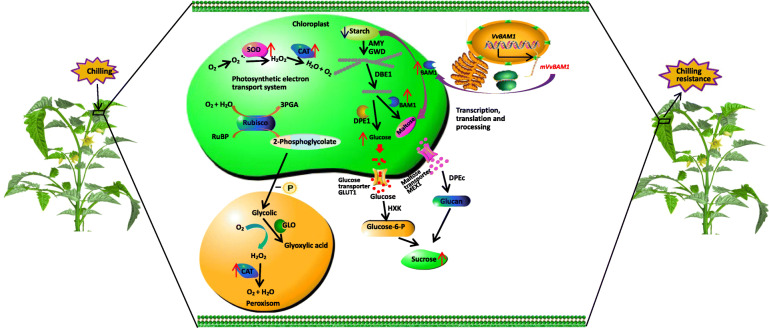
The overexpression of *VvBAM1* caused degradation of starch and increased the content of soluble sugar to maintain osmotic substances in the cytoplasm. Moreover, the change in sugar levels promotes elimination of reactive oxygen in tomatoes leaves, improving the tolerance to cold stress in tomato. Red arrows indicate increase, and blue arrows indicate decrease, respectively

The transport of sugars across membrane barriers greatly depends on sugar transporters, which catalyze through passive (but selective) diffusion or energy-dependent active transport [25, 58]. In leaves, starch hydrolysis starts with the phosphorylation of a small portion of the glucose residues of amylopectin by glucan-water dikinase (GWD) [17, 59]. Under LT stress, *VvBAM1* is rapidly transcribed in the nucleus, thereby leading to a rapid de novo BAM1 protein synthesis and an increased amylolytic activity (Fig. 7). Starch can be degraded directly by BAM or synergistically with α-amylase (AMY) and GWD to produce maltose. Amylose is produced under the enzymatic reaction of plastidic disproportionating enzyme 1; glucose and maltose are transported into the cytoplasm through glucose transporter 1 and maltose exporter 1; partially converted sucrose is used for osmotic stress regulation (Fig. 7) [23, 25, 60]. Transcriptome sequencing revealed that the sugar transport protein gene *SlSWEET4* and *SlSTP10* were upregulated by 2.9- and 18.6-fold in transgenic tomato plants under cold stress (Fig. 6b). This result was consistent with that of Nørholm et al. [61], who suggested that AtSTP10 can increase sugar contents in the cytoplasm in *A. thaliana*. These findings indicated that *BAM1* gene played an important role in regulating starch degradation and improving tolerance to LT stress.

## Conclusions

Overall, our results suggested that ectopic overexpression of *VvBAM1* can promote starch hydrolysis and increase sucrose and glucose contents in the cytoplasm, and significantly enhance the ROS scavenging by antioxidant enzymes, reducing the peroxidation effect of active oxygen on membrane lipid to protect the integrity of the cell membrane. BAMs have important research significance in plant response to abiotic stress, thereby helping further elucidate the role of amylase in improving plant resistance to LT stress.

## Methods

### Plant material and growth conditions

In this experiment, one-year-old grapevine (*Vitis amurensis* Rupr. *var.* ‘Zuoshan 1’) branches at different stages of development were subjected to qRT-PCR analysis to determine expression of the BAM genes family. The vineyard is located in Gansu Agricultural University (103.69 ° E, 36.09 ° N). We set five sampling stages as follow according to the lowest temperature change and the plant dormancy process as follows: the growth period is defined as A (20th Jul., the daily extreme temperatures is 18 °C to 33 °C); the early cold-hardening period is defined as B (17th Oct., 5 °C to 17 °C); the middle cold-hardening period is defined as C (28th Oct., 0 °C to 7 °C); the late cold-hardening period is defined as D (22th Nov., − 5 °C to 1 °C), and the deep dormancy period is defined as E (12th Jan., − 10 °C to 1 °C). Then, we collected the phloem of the grapevine branches, of which were frozen with liquid nitrogen and preserved at − 80 °C for RNA extraction and analysis of BAM genes expression. Tomato seeds (*Solanum lycopersicum* cv. ‘Micro-Tom’) were purchased from Nanjing Fengshuo Horiculture Ltd., Company (Nanjing, Jiangsu, China) and were used for *VvBAM1* transformation experiment. Positive transgenic tomato plants of T_0_ generation was transplanted into nutrient soil (ratio of vermiculite and culture soil is 1:3) to growth and collected seeds. The seeds of the transgenic tomato plants were harvested, dry naturally, and verified as transgenic materials. The positive transgenic tomato plants were used for subsequent LT stress tests. The WT tomato seeds were sown in a basic medium, and the seeds of the T_1_ generation transgenic tomato plants were sown in a medium containing hygromycin (50 mg/l) to further screen transgenic plants. The T_1_ generation seeds of hygromycin-resistant were collected to produce T_2_ generation transgenic materials. The same method was performed to produce T_3_ generation transgenic plants. The WT and T_3_ seeds were germinated in the dark at 27 °C for 3 d and then cultivated in individual culture soil pots (ratio of vermiculite and culture soil is 1:3) in growth chamber with conditions setting as follows: 27 °C/20 °C (16 h/8 h, light/dark) with light intensity of 280 μmol/m^2^ · s^− 1^, relative humidity was 70–80%. The six-week-old WT and T_3_ transgenic plants were subjected to LT stress at 6 °C. Final stress temperature was obtained by gradually decreasing temperature, and temperature gradient setting was from 27 °C to 14 °C, the decrease of 2 °C per 1 h, from 14 °C to 6 °C, the decrease of 1 °C per 1 h (Supplementary Fig. S5). Leaves, petioles, stems, and roots were collected for the analysis of tissues-specific expression and transcriptome sequencing of transgenic tomato plants at 6 °C for 24 h. Leaves from six-week-old tomato plants were utilized in all experiments and in three biological replicates.

### *VvBAM* genes family bioinformatics analysis

We obtained the accession numbers of the 9 *Arabidopsis thaliana* BAM genes referring to the research of Fulton et al. [17] and downloaded their full-length coding sequences (CDS) from data (https://www.arabidopsis.org/). Then, the 9 BAM gene sequences of *A. thaliana* were aligned with the grape genome database (http://www.genoscope.cns.fr/externe/GenomeBrowser/Vitis/) and obtained BAM gene family of grape. The BAM proteins sequence of apple (*M. domestica*), orange (*Cit.sinensis*), pear (*P. bretschneideri*), peach (*P. persica*), strawberry (*F. vesca*), and tomato (*Solanum lycopersicum*) were retrieved from Phytozome v12.1 (https://phytozome.jgi.doe.gov/pz/portal.html) and used to generate multiple sequence alignments in ClustalX v.2.0 with default settings. An unrooted phylogenetic tree of BAM protein from *Arabidopsis thaliana*, grape, apple, citrus, pear, peach, strawberry, and tomato was constructed using the maximum likelihood (ML) method through the MEGA 6.0 software with default setting and with bootstrap value of 1, 000. Multiple-sequence alignment and gene structure (exon and intron) analysis of *Arabidopsis thaliana* and grape were performed using the software DNAMAN 6.0 and the online website Gene Structure Display Serve GSDS v.2.0 (http://gsds.cbi.pku.edu.cn/) with parameters default settings. *Cis*-acting elements (2 k bp before the start codon of the gene) were analyzed using the PlantCARE database (http://bioinformatics.psb.ugent.be/webtools/plantcare/html/website) for prediction and plotted using TBtools software. The molecular weight and isoelectric point (pI) of grape BAM protein were predicted using EXPASY (http: //web.expasy.org/protparam/).

### RNA isolation and qRT-PCR analysis

The total RNA of grape branch was extracted using an OMEGA plant RNA kit (Omega Norcorss, GA, USA) according to the protocols. RNA samples (1 μg) were used for first-strand cDNA synthesized using PrimeScript™ IV 1st strand cDNA Synthesis Mix kit (TaKaRa, Japan) accordance with the manufacturer’s instructions. qPCR was performed using TB Green® Premix Ex Taq™ II (TaKaRa, Japan) and according to the manufacturer’s instruction. The reaction volume (20 μl) consist of 1 μl of cDNA (100 ng/μl), 10 μl of TB Green® Premix Ex Taq™ II, 2 μl of gene–specific primers (1 μl of forward and reverse primer, respectively), 7 μl of nuclease–free water. The qPCR program was initiated with a preliminary step of 1 min at 95 °C, followed by 40 cycles at 95 °C for 10 s, 55 °C for 30s and 72 °C for 20 s using Light Cycler®96 Real-Time PCR system (Roche, Switzerland). The primer was listed in Supplementary Table S1 that used for qPCR. The reference gene of grape was *VvGAPDH* (NCBI accession no. 973647) and data for each sample was calculated in relation to the reference gene using the 2^−ΔΔCT^ method [62].

### Gene clone and plasmid construction

The ORF of *VvBAM1* (gene accession no. XM_002285533, 1734 bp) was amplified using the grape cDNA as template. The forward primer GACACCCATGGTAGCAGCTATGAGTATCACCCATC; reverse primer GACACGGTCACCTCAGTGCATAAGAGCAACAGCAG. PCR amplification was performed as follows: at 95 °C for 5 min; 40 cycles at 94 °C for 30s, 58 °C for 30 s, 72 °C for 90 s; final extension at 72 °C for 5 min. The PCR product was detected via 1% agarose gel electrophoresis, before being linked into pGEM-T Easy Vector (Promega, Madison, WI, USA) for DNA sequencing (Supplementary Fig. S2a). The coding sequence of *VvBAM1* was cloned into *Nco* I and *BstE* II restriction sites of the binary vector pCAMBIA1301, behind the promoter of cauliflower mosaic virus *35S* (Supplementary Fig. S3b-c). The recombinant plasmid was transformed into *Agrobacterium tumefaciens* strain GV3101, and its presence was confirmed by PCR and sequencing analyses.

### Generation and identification of transgenic tomato

‘Micro Tom’ tomato plants were transformed via *Agrobacterium*–mediated leaf disk transformation in accordance with the methods described by Dan et al. and Hou et al. [63, 64]. In brief, 7d tomato in vitro plantlets (from seed germination) grown on basic medium (1/2 MS + 20 g/l sucrose + 6.5 g/l agar, pH 5.8–6.0), collecting the plant cotyledons, and remove the leaf tips and petioles. Cut a cotyledon into two explants with vertical veins, and spread them on pre-medium (1/2 MS + 20 g/l sucrose + 6.5 g/l agar + 1.0 mg/l kinetin, pH 5.8–6.0) to grow 2 d in the climate chamber (28 °C/20 °C, 16 h/8 h photoperiod, light/dark). A single colony *Agrobacterium* GV3101 with the recombinant plasmid was grown in LB medium (containing 50 mg/l rifampicin, 50 mg/l gentamicin, and 50 mg/l kanamycin) overnight at 28 °C shaking until the culture density reached an OD_600_ of 0.5–0.6. Then, the liquid medium with *Agrobacterium* was centrifuged at 5000 g to collect the bacteria. The *Agrobacterium* was suspended with 1/2 MS liquid medium with 100 mM acetosyringone to adjust an OD_600_ of 0.5–0.6. The pre-cultured explants were transferred into the suspension and incubated for 5 min away from light at room temperature. The explants were blot dried on sterilized filter paper to remove excess bacterial suspension, and then transferred to pre-medium co-cultivation and the plates were kept in dark at 28 °C. After 2 d, the explants were transferred to differentiation medium (MS + 20 g/l sucrose + 6.5 g/l agar + 2.0 mg/l zeatin + 30 mg/l hygromycin + 300 mg/l timetin). The explants were transferred to fresh differentiation medium every 15 d. When the explants started to differentiate, the differentiated buds were excised, and transferred to rooting medium (MS + 20 g/l sucrose + 6.5 g/l agar + 0.1 mg/l auxin + 15 mg/l hygromycin + 250 mg/l timentin). After rooting, the seedlings are transplanted into pots containing the substrate (ratio of vermiculite and culture soil is 1:3) (Fig. S2d-i). The genomic DNA was extracted from leaves using the CTAB (cetyltrimethylammonium bromide) method, and the transgenic tomato plants were identified with the cloning primer of VvBAM1 (Supplementary Fig. S2j). Primers were synthesized by Sangon Biotech (Shanghai, China), and their sequences for PCR were listed in Supplementary Table S1.

### Physiochemical analyses of transgenic tomato plants under LT

Leaves of WT and T_3_ tomato plants subjected to LT stress for 24 h at 6 °C were stained with nitroblue tetrazolium (NBT), 3, 3′-diaminobenzidine (DAB), and trypan blue to evaluate the accumulation of O_2_^−^, and H_2_O_2_, and cell damage were performed as described in Feng et al. [65] and Ma et al. [66]. Briefly, the treated leaves were immersed in 10 mM potassium phosphate buffer with 0.1% NBT (pH 7.8) and vacuum infiltrated for 5 min, and then the leaves were for 2 h at room temperature. After these steps, the leaves were boiled with NBT solution for 2 min. The tomato’s leaves were immersed in aqueous solution with 1 mg/ml DAB (pH 7.0), and then incubated for 8 h at room temperature in the dark, then boiling water bath for 5 min. The after staining leaves of tomato plants were decolorized with absolute ethanol for 2 h. The decolorizing solution (ethanol: lactic acid: glycerin, 3: 1: 1) was used to decolorize the leaves after dyeing with NBT and DAB until no longer contained chlorophyll. The tomato leaves from treatment were boiling trypan blue solution for 5 min, and then using the 2.5 g/ml chloral hydrate decolorizing for 2 h. Leaves of WT and T_3_ transgenic plants were harvested after 0, 3, 6, 12, 24, 48 and 72 h points at 6 °C stress for electrolyte leakage assay. Electrolyte leakage was measured according to the methods of Ma et al. [66]. Briefly, three full expanded leaves at the six-week-old from WT and T_3_ transgenic plants were cut into a disc shape (1 cm diameter) and immersed in 15 ml of double distilled water in a 30 ml glass tube for 24 h with shaking. The initial conductivity (C_1_) was measured with a model DDS-307A device (Shanghai Leici Instrument). Then, the tube with leaf disc shape was boiled and the conductivity was determined (C_2_). The relative electrolyte leakage was calculated as the ratio of C_1_ to C_2_. Total amylase and BAM activities, POD, SOD, CAT, starch, glucose, and sucrose contents were determined using a commercial chemical assay kit (Jiangsu Keming Biotechnology Institute, Suzhou, China) according to the manufacturer’s instructions. Leaf sample weight for biochemical indicator detection was 0.2 g. Three independent biological replicates were analyzed in all experiments.

### Different tissue expression levels of *VvBAM1* in transgenic plants under LT stress

The tissue-specific expression of the gene elucidates potential plant relationship because the expression of a certain gene and its function in different organs can be observed. Therefore, the leaf, petiole, stem, and root of the six-week-old WT and three transgenic plants were selected to analyze the expression level of *VvBAM1* under normal growth and LT stress (6 °C). The leaf, petiole, stem, and root samples were collected, frozen in liquid nitrogen, and stored at − 80 °C. RNA extraction and cDNA synthesis were conducted as described above. All qRT-PCR primers were designed using Primer3 Input (http://primer3.ut.ee/), and data for each sample were calculated in relation to the reference gene *SlActin* (NCBI accession no. NM_001330119).

### RNA-seq library preparation and sequencing

Effect of overexpression of *VvBAM1* on the expression of genes related to LT stress was analyzed using transcription profile. RNA extraction and cDNA synthesis of the transgenic tomato plants were conducted as described above. Transcriptome sequencing of WT and T_3_ generation tomato leaves under stress at 6 °C for 24 h was commissioned by Biomarker Technologies Co., Ltd. (Beijing, China). RNA quality and concentration were measured by agarose gel electrophoresis through NanoPhotometer spectrophotometer (Implen, Germany) and Agilent 2100 BioAnalyzer (Agilent Technologies, USA), respectively. The cDNA of about 250–300 bp was screened with AMPure XP beads. PCR amplification products were repurified with AMPure XP beads to obtain the library. A Qubit 2.0 fluorometer (Invitrogen, USA) was used for preliminary quantification. The library was diluted to 1.5 ng/μl, and the insert size of the library was detected via the Agilent 2100 BioAnalyzer. Then, the Novaseq6000 platform sequencing was performed. All experiments were conducted with three independent biological replicates. The differentially expressed genes (DEGs) were identified through false discovery rate (FDR) < 0.05 and |Log_2_ FC (fold-change)| ≥ 2 as thresholds screening. After sequencing, a total of 38.17 Gb of clean reads by removing sequencing adapters and low-quality reads, more than 92% reads had a quality score of Q30 (sequencing error rate, 0.1%). The sequencing data results are listed in Table S3 (Supplementary Table S3).

### Statistical analysis

All experiment data were presented means ± standard deviation (SD). Statistical analysis was performed via one-way ANOVA. Significant different were evaluated by Duncan’s tests using SPSS 22.0 software (SPSS Inc., Chicago, USA), which are indicated by lowercase (*P* < 0.05).

## Supplementary Information

**Additional file 1: Supplementary Table S1.** qRT-PCR primers for expression on analysis of *VvBAM* genes.

**Additional file 2: Supplementary Table S2.** Information of the *BAM* genes used to construct the phylogenetic tree in Fig. 1a.

**Additional file 3: Supplementary Table S3.** Summary of reads based on RNA sequence data obtained from each sample after 24 h LT stress.

**Additional file 4: Supplementary Table S4.** Annotation information of DEGs in heatmaps.

**Additional file 5: Supplementary Fig. S1.** Analysis of BAM gene motif in Fig. 1a.

**Additional file 6: Supplementary Fig. S2.** The *VvBAM1* was cloned and tomato transformation. **a** Electrophoretic analysis of *VvBAM1* gene fragement PCR amplification. The red box indicates amplificated *VvBAM1* products. M stands for DNA maker ladder (DL 5000 bp). **b** Electrophoretic analysis of recombinant vector pCAMBIA1301-*VvBAM1* was confirmed by double restriction enzyme digestion with *Nco I* and *BstE II*. E stands for recombinant vector by enzyme digestion. M stands for DNA maker ladder (DL 10kbp). **c** *VvBAM1* was inserted into the *Nco I* and *BstE* II site of the pCAMBIA1301 vector. **d** – **i** Growth and differentiation of tomato explant. **j** The resulting transgenic plants were identified individually by PCR. The red box indicates that the results of screening of transgenic tomato plants. M stands for DNA maker ladder (DL 2000 bp). P stands for positive control. WT stands for negative control. OE-1 – OE-4 stand for four transgenic tomato plants.

**Additional file 7: Supplementary Fig. S3.** Six-week-old WT and transgenic tomato plants. **a** Phenotype of six-week-old WT and transgenic plants. **b** The roots growth of six-week-old WT and transgenic tomato plants.

**Additional file 8: Supplementary Fig. S4.** The plant height of six-week-old WT and transgenic tomato plants were analyzed.

**Additional file 9: Supplementary Fig. S5.** The changes of temperature during the process of LT stress. Changes trend: a decrease of 2 °C per 1 h from 28 °C to 14 °C, followed by a decrease of 1 °C per 1 h from 14 °C to 6 °C, determining and analyzing the optimal sample collection time point at 6 °C LT stress for 72 h. The red segment is the final sampling time interval.

**Additional file 10: Supplementary Fig. S6.** Analysis of relative electrolyte leakage and expression of *VvBAM1* under different stress time points in WT and transgenic plants leaves. **a** Relative electrolyte leakage of tomato leaves under different stress time points. **b** Relative expression level of *VvBAM1* under different stress time points in tomato leaves.

## Data Availability

All data generated or analysed during this study are included in supplementary information files. The RNA-sequencing data have been deposited with NCBI (https://submit.ncbi.nlm.nih.gov/subs/sra/) under BioProject PRJNA703431.

## Abbreviations

BAM: β-Amylase
WT: Wild type plants
POD: Peroxidase
SOD: Superoxide dismutase
CAT: Catalase
DAB: 3, 3′-diaminobenzidine
NBT: Nitroblue tetrazolium
DEGs: Differentially expressed genes
ROS: Reactive oxygen species
AMY: α-Amylase
Jul.: July
Oct.: October
Nov.: November
Jan.: January
NCBI: National center for biotechnology information
PCR: Polymerase chain reaction
qRT-PCR: Quantitative real-time PCR
FDR: False discovery rate
FC: Fold change
h: Hour
CaMV35S: Cobacco cauliflower mosaic virus
GWD: Glucan-water dikinase
