# Supplementary material for: Genome-wide identification of BAM genes in grapevine (Vitis vinifera L.) and ectopic expression of VvBAM1 modulating soluble sugar levels to improve low-temperature tolerance in tomato
Source: BMC Plant Biol. 2021 Mar 26;21:156. doi: 10.1186/s12870-021-02916-8 (PMC8004407; doi:10.1186/s12870-021-02916-8)
Supplement: Supplementary file 1 — Additional file 1: Supplementary Table S1. qRT-PCR primers for expression on analysis of VvBAM genes. [file 12870_2021_2916_MOESM1_ESM.docx]

| Table S1 qRT-PCR primers for expressin on analysis of *VvBAM*s | | | | |
| --- | --- | --- | --- | --- |
| Gene name | Gene accession NO. | qPCR Primer 5＇→3＇ | | Product size (bp) |
| *VvBAM1* | GSVIVT01001863001 | F: GTTGCCGCTTCCGCCCATAC | R: CCGCCCTCATCCCTCCCATC | 104 |
| *VvBAM2* | GSVIVT00026862001 | F: GGGCTGCTCTGAACTTCACCTG | R: TGGGTCTGCCAGTGCCTCTG | 80 |
| *VvBAM3* | GSVIVT00015861001 | F: TGTGACTCTAGCGGGACAGACC | R: CACAAGAGCAGCCTCCATCGTC | 84 |
| *VvBAM4* | GSVIVT00026081001 | F: GCTCGGGCATGGTCTGTCTTTC | R: TGGAGCCTCTGCTGCTGTGG | 94 |
| *VvBAM5* | GSVIVT00026861001 | F: TGGGAGATAGAGGAGGGCAAGC | R: AACTTCGGCGGCATGTCAACC | 97 |
| *VvBAM6* | GSVIVT00001210001 | F: CCTCAGCCTCAGCCTCAGTCTC | R: GGTCCGCTCCTTCTCCTTCTCC | 144 |
| *VvGAPDH* | XM_0022663109 | F: TTCTCGTTGAGTGCTATTCCA | R: CCACAGACTTCATCGGTGACA | 70 |
| *SlActin* | NM_001330119 | F: GGCAGACGGAGAGGATATTCA | R: TGACCCATACCCACCATCAC | 150 |
