# Supplementary material for: Genome-wide identification of BAM genes in grapevine (Vitis vinifera L.) and ectopic expression of VvBAM1 modulating soluble sugar levels to improve low-temperature tolerance in tomato
Source: BMC Plant Biol. 2021 Mar 26;21:156. doi: 10.1186/s12870-021-02916-8 (PMC8004407; doi:10.1186/s12870-021-02916-8)
Supplement: Supplementary file 2 — Additional file 2: Supplementary Table S2. Information of the BAM genes used to construct the phylogenetic tree in Fig. 1a. [file 12870_2021_2916_MOESM2_ESM.docx]

Table S2 Information of the *BAM* genes used to construct the phylogenetic tree in Fig. 1a

| Plants species | Genes | Gene ID |
| --- | --- | --- |
| *Citrus sinensis* | *Cit. sinensis BAM1* | XM_006420353.2 |
|  | *Cit. sinensis BAM8* | XM_006494044.3 |
|  | *Cit. sinensis BAM9* | XM_006489097.3 |
|  | *Cit. sinensis BAM2* | XM_006491033.3 |
|  | *Cit. sinensis BAM3* | XM_006440076.2 |
|  | *Cit. sinensis BAM7* | XM_006491031.3 |
|  | *Cit. sinensis BAM4* | XM_006451012.3 |
|  | *Cit. sinensis BAM5* | XM_006469669.3 |
| *Prunus persica* | *P. persica BAM1* | XM_007223052.2 |
|  | *P. persica BAM9* | XM_007222426.2 |
|  | *P. persica BAM3-1* | XM_007209805.2 |
|  | *P. persica BAM3-2* | XM_007209028.2 |
|  | *P. persica BAM7* | XM_007220161.2 |
|  | *P. persica BAM8* | XM_007210766.6 |
|  | *P. persica BAM2* | XM_007217996.2 |
|  | *P. persica BAM5* | XM_007215060.2 |
|  | *P. persica BAM4* | XM_007211535.2 |
| *Solanum lycopersicum* | *S. lycopersicum BAM1* | NM_001247627.2 |
|  | *S. lycopersicum BAM7* | XM_006360516.2 |
|  | *S. lycopersicum BAM2* | XM_004245434.3 |
|  | *S. lycopersicum BAM3* | XM_004245796.4 |
|  | *S. lycopersicum BAM8* | XM_004244394.4 |
|  | *S. lycopersicum BAM5* | XM_004233291.4 |
|  | *S. lycopersicum BAM9* | NM_001247123.2 |
|  | *S. lycopersicum BAM6* | XM_004249364.4 |
|  | *S. lycopersicum BAM4* | XM_004743400.3 |
| *Malus domestica* | *M. domestica BAM9* | XM_008392519.3 |
|  | *M. domestica BAM8* | XM_008375215.3 |
|  | *M. domestica BAM7* | XM_029105701.1 |
|  | *M. domestica BAM1* | XM_008344331.3 |
|  | *M. domestica BAM2* | XM_008340636.3 |
|  | *M. domestica BAM5* | XM_008381376.3 |
|  | *M. domestica BAM6* | XM_029094710.1 |
|  | *M. domestica BAM3* | XM_008375497.3 |
| *Arabidopsis thaliana* | *A. thaliana BAM1* | AT3G23920 |
|  | *A. thaliana BAM2* | AT4G00490 |
|  | *A. thaliana BAM3* | AT4G17090 |
|  | *A. thaliana BAM4* | AT5G55700 |
|  | *A. thaliana BAM5* | AT4G15210 |
|  | *A. thaliana BAM6* | AT2G32290 |
|  | *A. thaliana BAM7* | AT2G45880 |
|  | *A. thaliana BAM8* | AT5G45300 |
|  | *A. thaliana BAM9* | AT5G18670 |
| *Fragaria vesca* | *F. vesca BAM7* | XM_004306739.2 |
|  | *F. vesca BAM8* | XM_011466987.1 |
|  | *F. vesca BAM1* | XM_004296501.2 |
|  | *F. vesca BAM3* | XM_004300249.2 |
|  | *F. vesca BAM4* | XM_004291761.2 |
|  | *F. vesca BAM9* | XM_004296745.2 |
| *Pyrus bretschneideri* | *P. bretschneideri BAM3* | XM_009373582.2 |
|  | *P. bretschneideriBAM7* | XM_009342147.2 |
|  | *P. bretschneideriBAM8* | XM_018649498.1 |
|  | *P. bretschneideriBAM1* | XM_009351836.2 |
|  | *P. bretschneideriBAM4* | XM_018651561.1 |
|  | *P. bretschneideriBAM9* | XM_009370828.2 |
|  | *P. bretschneideriBAM2* | XM_009353236.2 |
| *Vitis vinifera* | *V. vinifera BAM1* | GSVIVT01026922001 |
|  | *V. vinifera BAM2* | GSVIVT01026920001 |
|  | *V. vinifera BAM3* | GSVIVT01013272001 |
|  | *V. vinifera BAM4* | GSVIVT01001863001 |
|  | *V. vinifera BAM5* | GSVIVT01030642001 |
|  | *V. vinifera BAM6* | GSVIVT01036911001 |
