# Supplementary material for: Genome-wide identification of BAM genes in grapevine (Vitis vinifera L.) and ectopic expression of VvBAM1 modulating soluble sugar levels to improve low-temperature tolerance in tomato
Source: BMC Plant Biol. 2021 Mar 26;21:156. doi: 10.1186/s12870-021-02916-8 (PMC8004407; doi:10.1186/s12870-021-02916-8)
Supplement: Supplementary file 3 — Additional file 3: Supplementary Table S3. Summary of reads based on RNA sequence data obtained from each sample after 24 h LT stress. [file 12870_2021_2916_MOESM3_ESM.docx]

Table S3 Summary of reads based on RNA sequence data obtained from each sample after 24h cold stress.

| Samples | Clean reads | Clean bases | GC Content (%) | % ≥ Q30 |
| --- | --- | --- | --- | --- |
| WT1 | 24,130,972 | 7,212,984,404 | 43.79% | 93.21% |
| WT2 | 21,525,091 | 6,430,632,794 | 43.72% | 92.42% |
| WT3 | 21,045,964 | 6,290,772,322 | 44.55% | 92.27% |
| OE1 | 21,397,526 | 6,390,246,946 | 43.73% | 92.67% |
| OE2 | 23,841,237 | 7,120,754,996 | 43.82% | 92.35% |
| OE3 | 25,819,272 | 7,711,854,296 | 43.77% | 92.86% |

% ≥ Q30: The percentage of clean reads whose quality score was more than 30.
