# Supplementary material for: Genome-wide identification of BAM genes in grapevine (Vitis vinifera L.) and ectopic expression of VvBAM1 modulating soluble sugar levels to improve low-temperature tolerance in tomato
Source: BMC Plant Biol. 2021 Mar 26;21:156. doi: 10.1186/s12870-021-02916-8 (PMC8004407; doi:10.1186/s12870-021-02916-8)
Supplement: Supplementary file 4 — Additional file 4: Supplementary Table S4. Annotation information of DEGs in heatmaps. [file 12870_2021_2916_MOESM4_ESM.docx]

Table S4 The DEGs and annotation information of heatmaps.

| Functional category | Gene ID | Annotation |
| --- | --- | --- |
| Sugar transport protein | Solyc01g080680.3 | Sugar transporter ERD6-like 16 GN=At5g18840 PE=2 SV=2 |
|  | Solyc10g062085.2 | Sugar transporter ERD6-like 8 GN=At3g05150 PE=2 SV=1 |
|  | Solyc10g062090.1 | Sugar transporter ERD6-like 7 GN=At2g48020 PE=2 SV=2 |
|  | Solyc10g062110.2 | Sugar transporter ERD6-like 8 GN=At3g05150 PE=2 SV=1 |
|  | Solyc02g062750.4 | Sugar transporter ERD6-like 6 GN=At1g75220 PE=1 SV=1 |
|  | Solyc02g071520.3 | Bidirectional sugar transporter SWEET2a GN=SWEET2A PE=2 SV=1 |
|  | Solyc02g085180.5 | Bidirectional sugar transporter SWEET2a GN=SWEET2A PE=2 SV=1 |
|  | Solyc01g008240.4 | Sugar transport protein 10 GN=STP10 PE=2 SV=1 |
|  | Solyc03g005880.3 | Bidirectional sugar transporter SWEET2a GN=SWEET2A PE=2 SV=1 |
|  | Solyc03g097570.3 | Bidirectional sugar transporter SWEET11 OS=Arabidopsis thaliana OX=3702 GN=SWEET11 PE=1 SV=1 |
|  | Solyc03g097580.4 | Bidirectional sugar transporter SWEET10 GN=SWEET10 PE=1 SV=1 |
|  | Solyc05g024260.3 | Bidirectional sugar transporter N3 GN=N3 PE=2 SV=1 |
|  | Solyc05g052790.3 | Probable sugar phosphate/phosphate translocator At3g11320 GN=At3g11320 PE=2 SV=1 |
|  | Solyc06g060590.3 | Bidirectional sugar transporter SWEET1 GN=SWEET1 PE=1 SV=1 |
|  | Solyc06g071400.3 | Bidirectional sugar transporter SWEET5 GN=SWEET5 PE=1 SV=2 |
|  | Solyc09g075820.3 | Sugar transport protein 13 GN=STP13 PE=1 SV=2 |
|  | Solyc01g098500.3 | Sugar transporter ERD6-like 5 GN=At1g54730 PE=2 SV=2 |
|  | Solyc01g098560.3 | Sugar transporter ERD6-like 5 GN=At1g54730 PE=2 SV=2 |
|  | Solyc01g099880.4 | Bidirectional sugar transporter SWEET16 GN=SWEET16 PE=3 SV=1 |
|  | Solyc02g005180.3 | Sugar transporter ERD6-like 5 GN=At1g54730 PE=2 SV=2 |
|  | Solyc02g079220.4 | Sugar carrier protein C GN=STC PE=2 SV=1 |
|  | Solyc03g078600.3 | Sugar transport protein 1 GN=STP1 PE=1 SV=2 |
|  | Solyc03g097600.3 | Bidirectional sugar transporter SWEET10 GN=SWEET10 PE=1 SV=1 |
|  | Solyc03g097610.3 | Bidirectional sugar transporter SWEET10 GN=SWEET10 PE=1 SV=1 |
|  | Solyc03g097870.4 | Bidirectional sugar transporter SWEET11 GN=SWEET11 PE=1 SV=1 |
|  | Solyc04g064610.3 | Bidirectional sugar transporter SWEET1 GN=SWEET1 PE=1 SV=1 |
|  | Solyc04g064640.4 | Bidirectional sugar transporter SWEET1 GN=SWEET1 PE=1 SV=1 |
|  | Solyc04g080460.3 | Sugar transporter ERD6-like 6 GN=At1g75220 PE=1 SV=1 |
|  | Solyc06g054270.3 | Sugar transport protein 8 GN=STP8 PE=2 SV=2 |
|  | Solyc06g072630.4 | Bidirectional sugar transporter SWEET12 GN=SWEET12 PE=1 SV=1 |
|  | Solyc06g072640.1 | Bidirectional sugar transporter SWEET11 GN=SWEET11 PE=1 SV=1 |
| POD protein | Solyc07g052550.2 | Peroxidase 3 GN=PER3 PE=2 SV=1 |
|  | Solyc09g072700.4 | Peroxidase 44 GN=PER44 PE=2 SV=1 |
|  | Solyc10g047110.2 | Peroxidase 43 GN=PER43 PE=3 SV=2 |
|  | Solyc10g076190.2 | Peroxidase 4 GN=PER4 PE=1 SV=1 |
|  | Solyc11g007220.2 | Peroxidase 2 GN=PER2 PE=2 SV=1 |
|  | Solyc11g010120.2 | Peroxidase 17 GN=PER17 PE=2 SV=1 |
|  | Solyc12g096530.1 | Peroxidase 46 GN=PER46 PE=3 SV=1 |
|  | Solyc01g104860.3 | Peroxidase 43 GN=PER43 PE=3 SV=2 |
|  | Solyc02g062510.3 | Peroxidase 72 GN=PER72 PE=1 SV=1 |
|  | Solyc05g055320.3 | Peroxidase 5 GN=PER5 PE=1 SV=2 |
|  | Solyc01g058520.4 | Peroxidase 40 GN=PER40 PE=2 SV=2 |
|  | Solyc01g101050.3 | Peroxidase 5 GN=PER5 PE=1 SV=2 |
|  | Solyc02g084800.4 | Peroxidase 72 GN=PER72 PE=1 SV=1 |
|  | Solyc04g080760.3 | Peroxidase 9 GN=PER9 PE=1 SV=1 |
|  | Solyc05g052280.3 | Peroxidase 52 GN=PER52 PE=2 SV=1 |
|  | Solyc06g050440.3 | Peroxidase 4 GN=PER4 PE=1 SV=1 |
| SOD protein | Solyc03g062890.3 | Superoxide dismutase [Cu-Zn] 2 GN=SODCC.2 PE=2 SV=1 |
|  | Solyc03g095180.3 | Superoxide dismutase [Fe] 2, chloroplastic GN=FSD2 PE=1 SV=1 |
|  | Solyc06g048410.4 | Superoxide dismutase [Fe], chloroplastic (Fragment) GN=SODB PE=2 SV=1 |
|  | Solyc01g067740.3 | Superoxide dismutase [Cu-Zn] 2 GN=SODCC.5 PE=3 SV=3 |
|  | Solyc06g049080.3 | Superoxide dismutase [Mn], mitochondrial GN=SODA PE=1 SV=1 |
| CAT protein | Solyc01g100640.4 | Catalase isozyme 1 (Fragment) GN=CAT1 PE=2 SV=1 |
|  | Solyc04g082460.4 | Catalase isozyme 3 GN=CAT3 PE=2 SV=1 |
|  | Solyc04g082475.1 | Catalase isozyme 3 GN=CAT3 PE=2 SV=1 |
|  | Solyc12g094620.3 | Catalase isozyme 1 GN=CAT1 PE=2 SV=1 |
|  | Solyc02g082760.3 | Catalase isozyme 2 GN=CAT2 PE=2 SV=1 |
