# Supplementary figures and images for: Genome-wide identification of BAM genes in grapevine (Vitis vinifera L.) and ectopic expression of VvBAM1 modulating soluble sugar levels to improve low-temperature tolerance in tomato
Source: BMC Plant Biol. 2021 Mar 26;21:156. doi: 10.1186/s12870-021-02916-8 (PMC8004407; doi:10.1186/s12870-021-02916-8)

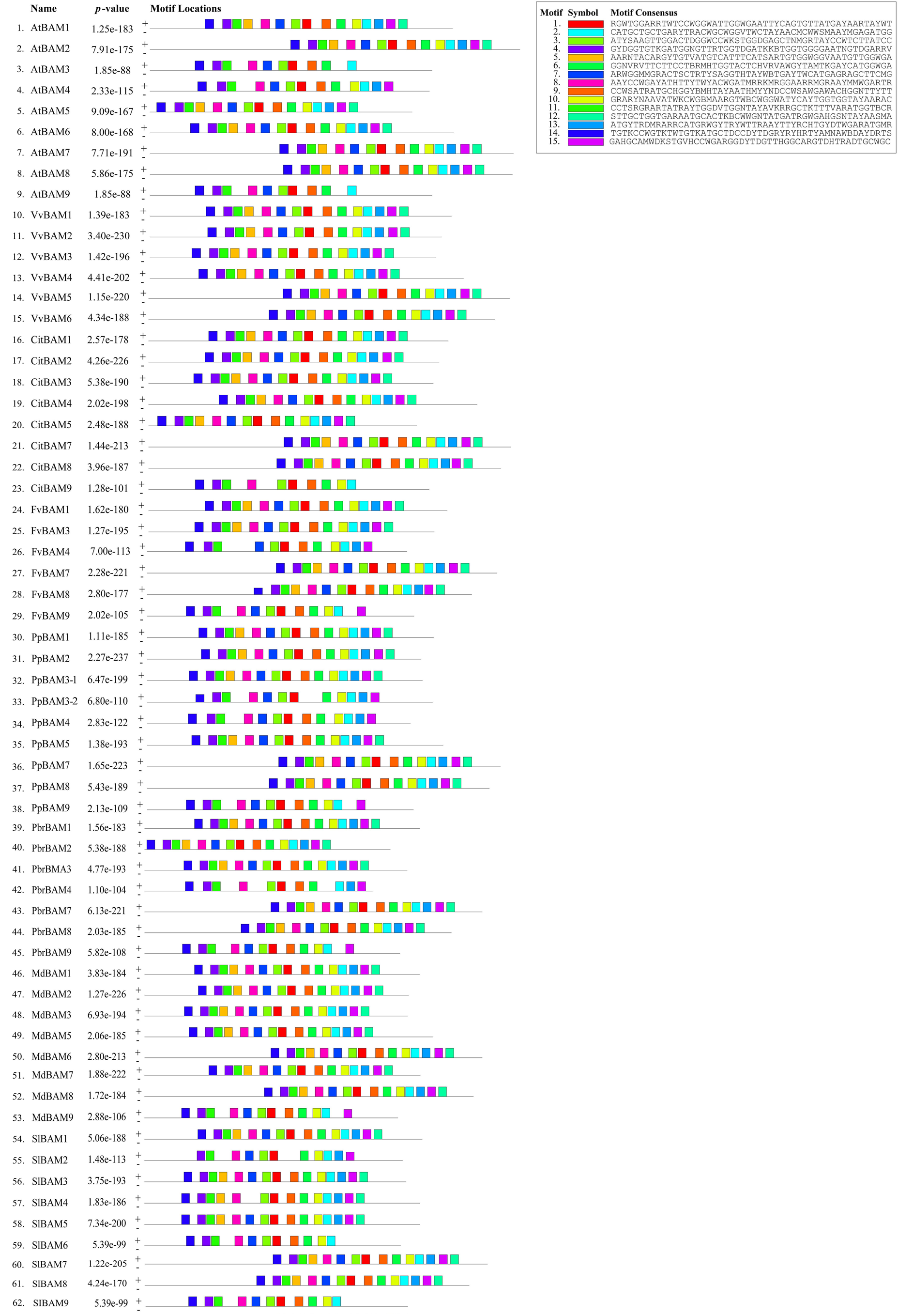

Supplement: Supplementary file 5 — Additional file 5: Supplementary Fig. S1. Analysis of BAM gene motif in Fig. 1a. [file 12870_2021_2916_MOESM5_ESM.tif]

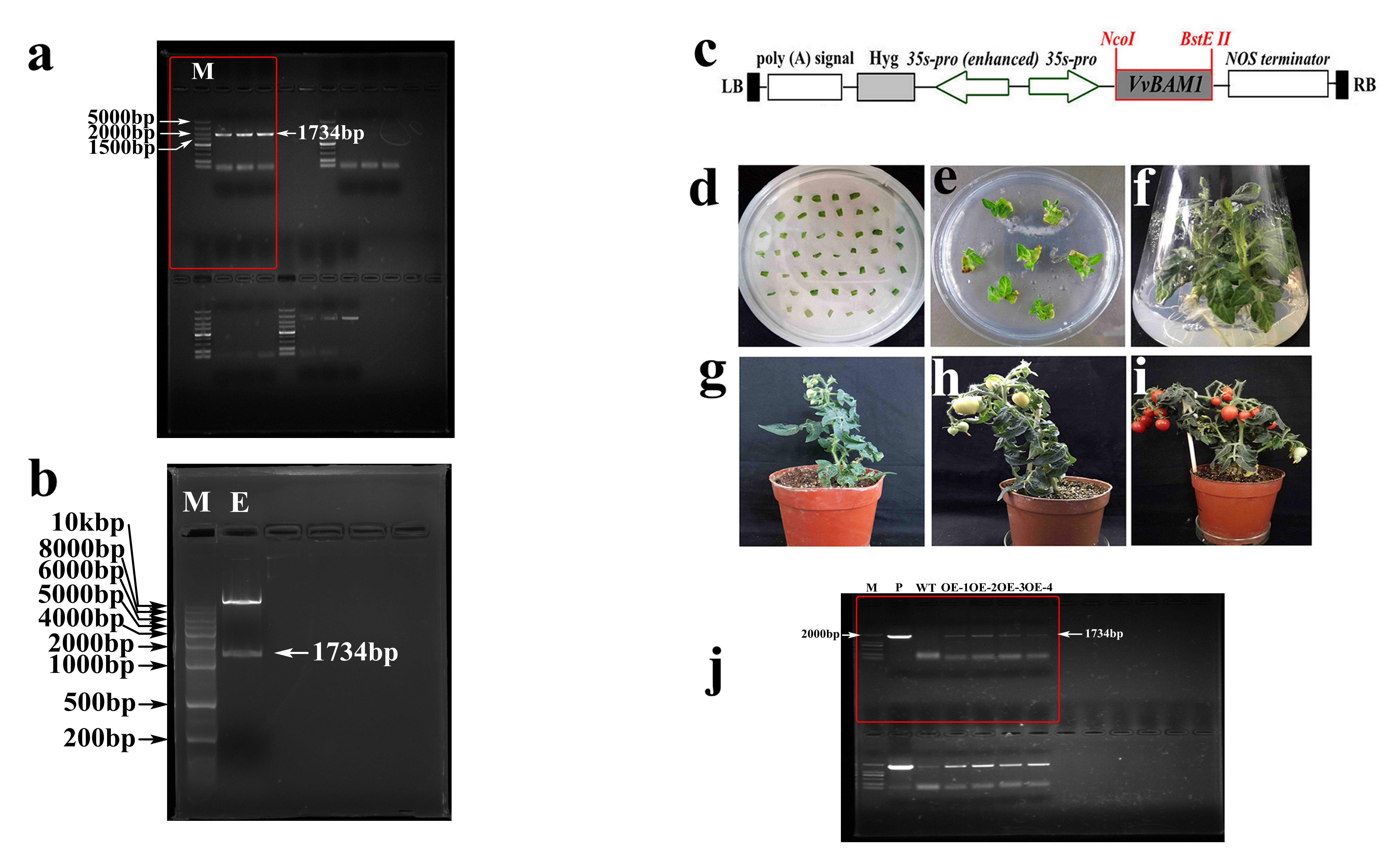

Supplement: Supplementary file 6 — Additional file 6: Supplementary Fig. S2. The VvBAM1 was cloned and tomato transformation. a Electrophoretic analysis of VvBAM1 gene fragement PCR amplification. The red box indicates amplificated VvBAM1 products. M stands for DNA maker ladder (DL 5000 bp). b Electrophoretic analysis of recombinant vector pCAMBIA1301-VvBAM1 was confirmed by double restriction enzyme digestion with Nco I and BstE II. E stands for recombinant vector by enzyme digestion. M stands for DNA maker ladder (DL 10kbp). c VvBAM1 was inserted into the Nco I and BstE II site of the pCAMBIA1301 vector. d – i Growth and differentiation of tomato explant. j The resulting transgenic plants were identified individually by PCR. The red box indicates that the results of screening of transgenic tomato plants. M stands for DNA maker ladder (DL 2000 bp). P stands for positive control. WT stands for negative control. OE-1 – OE-4 stand for four transgenic tomato plants. [file 12870_2021_2916_MOESM6_ESM.tif]

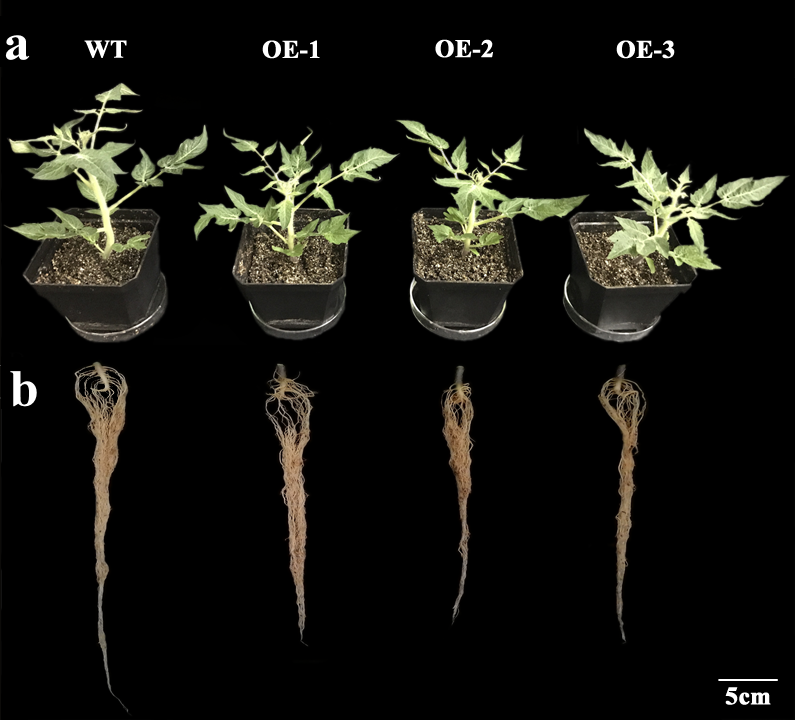

Supplement: Supplementary file 7 — Additional file 7: Supplementary Fig. S3. Six-week-old WT and transgenic tomato plants. a Phenotype of six-week-old WT and transgenic plants. b The roots growth of six-week-old WT and transgenic tomato plants. [file 12870_2021_2916_MOESM7_ESM.tif]

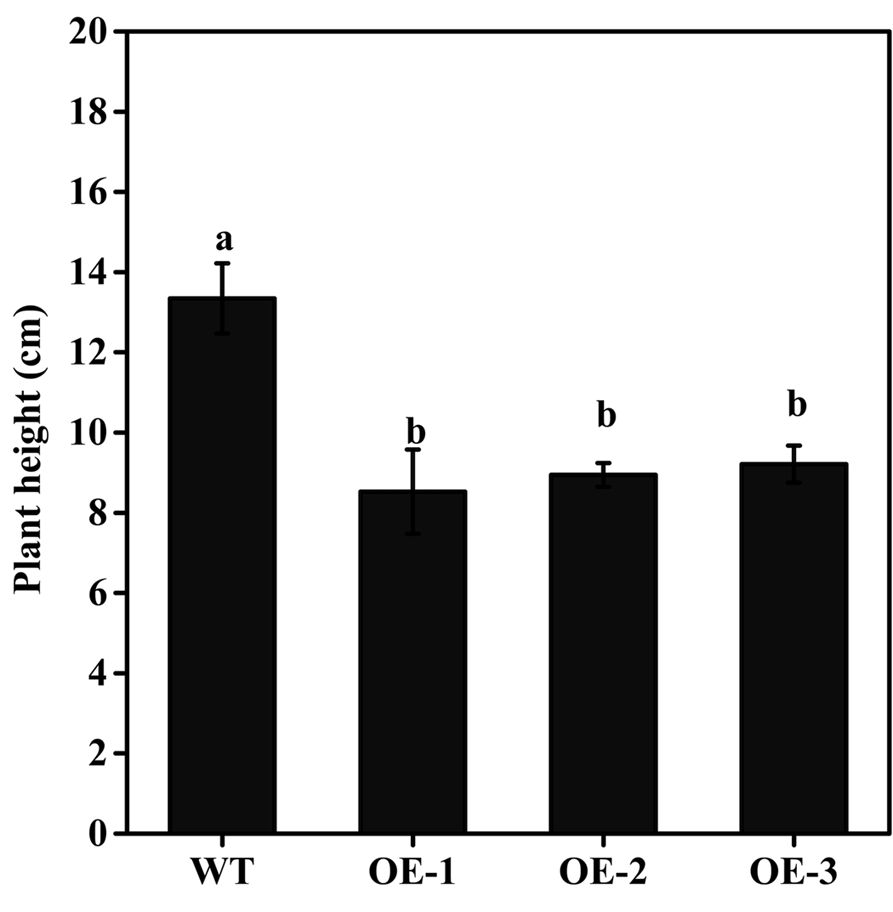

Supplement: Supplementary file 8 — Additional file 8: Supplementary Fig. S4. The plant height of six-week-old WT and transgenic tomato plants were analyzed. [file 12870_2021_2916_MOESM8_ESM.tif]

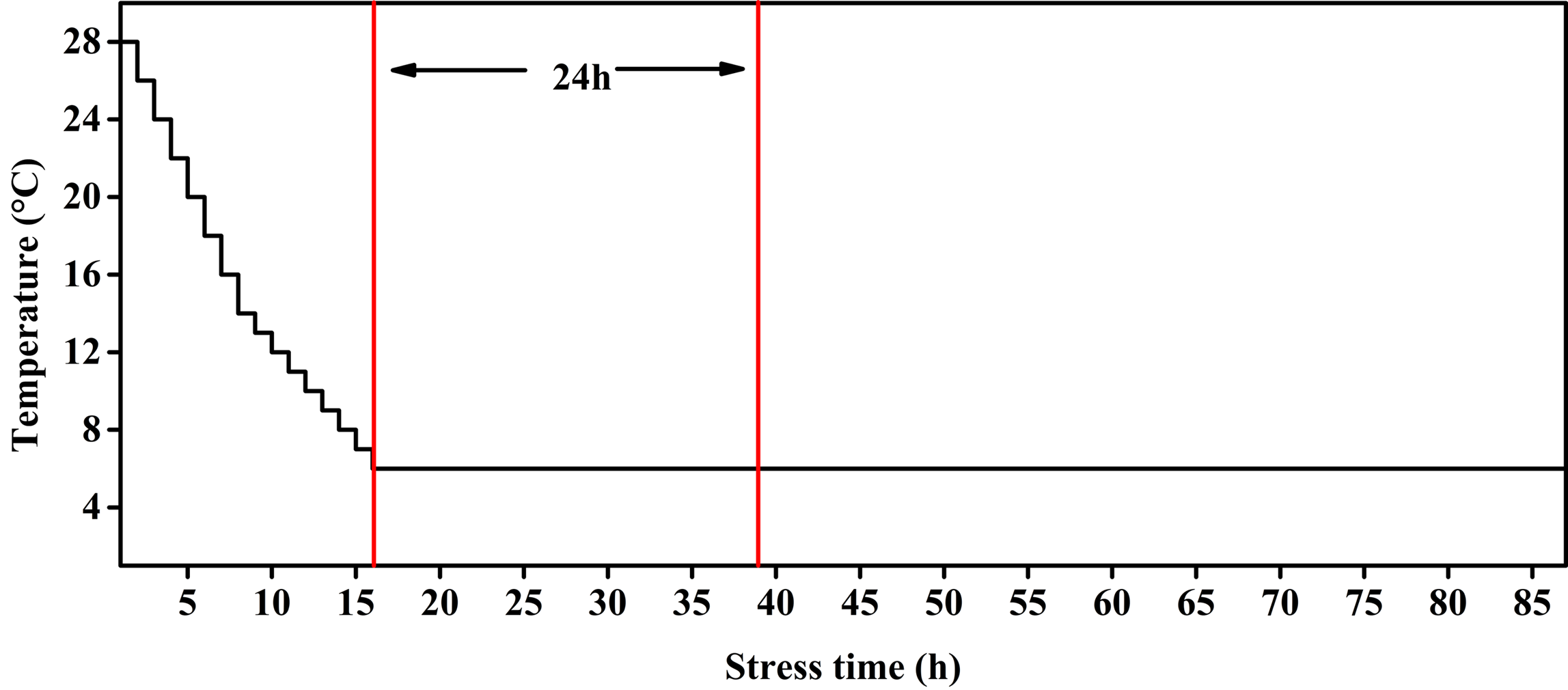

Supplement: Supplementary file 9 — Additional file 9: Supplementary Fig. S5. The changes of temperature during the process of LT stress. Changes trend: a decrease of 2 °C per 1 h from 28 °C to 14 °C, followed by a decrease of 1 °C per 1 h from 14 °C to 6 °C, determining and analyzing the optimal sample collection time point at 6 °C LT stress for 72 h. The red segment is the final sampling time interval. [file 12870_2021_2916_MOESM9_ESM.tif]

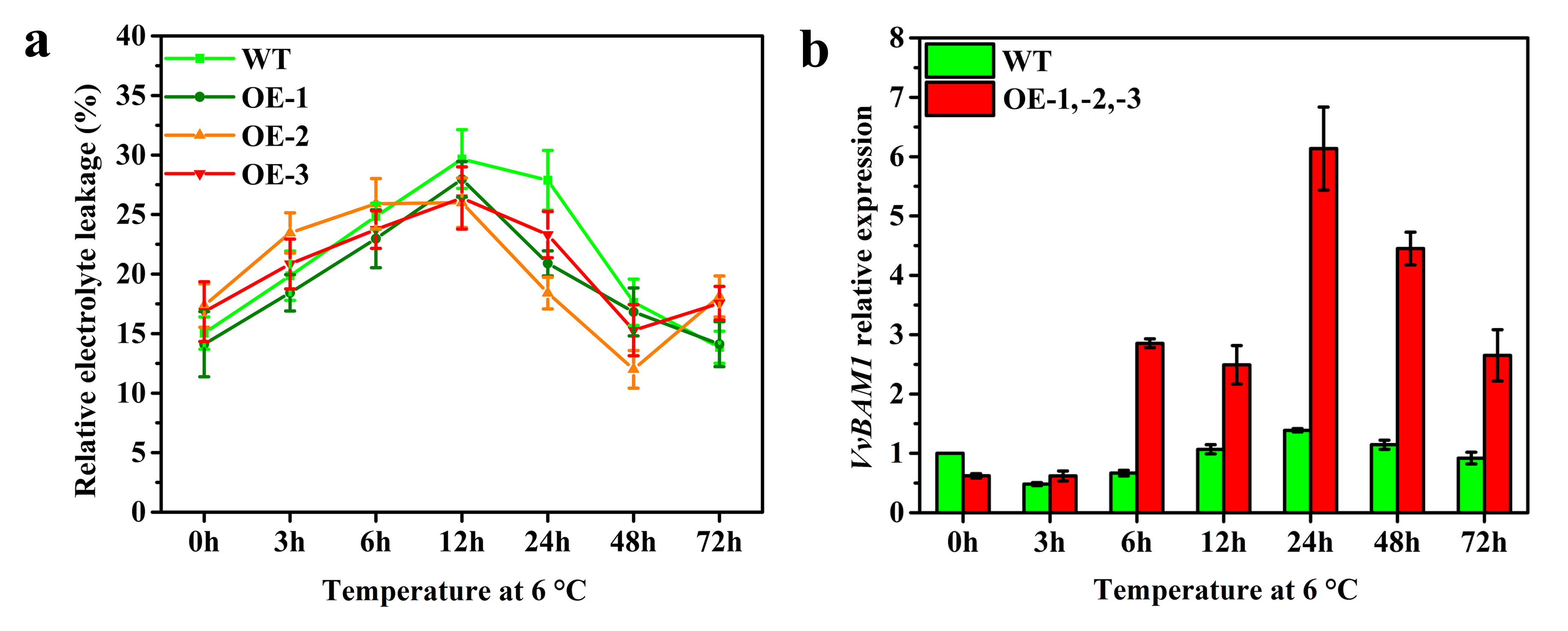

Supplement: Supplementary file 10 — Additional file 10: Supplementary Fig. S6. Analysis of relative electrolyte leakage and expression of VvBAM1 under different stress time points in WT and transgenic plants leaves. a Relative electrolyte leakage of tomato leaves under different stress time points. b Relative expression level of VvBAM1 under different stress time points in tomato leaves. [file 12870_2021_2916_MOESM10_ESM.tif]
